# *LRRK2* Gene Variants Associated With a Higher Risk for Alcohol Dependence in Multiethnic Populations

**DOI:** 10.3389/fpsyt.2021.665257

**Published:** 2021-05-31

**Authors:** Pablo Rafael Silveira Oliveira, Lorena Oliveira de Matos, Nathalia Matta Araujo, Hanaísa P. Sant Anna, Daniel Almeida da Silva e Silva, Andresa K. Andrade Damasceno, Luana Martins de Carvalho, Bernardo L. Horta, Maria Fernanda Lima-Costa, Mauricio Lima Barreto, Corinde E. Wiers, Nora D. Volkow, Ana Lúcia Brunialti Godard

**Affiliations:** ^1^Instituto de Biologia, Universidade Federal da Bahia, Salvador, Brazil; ^2^Centro de Integração de Dados e Conhecimentos para Saúde, Fundação Oswaldo Cruz, Salvador, Brazil; ^3^Departamento de Genética, Ecologia e Evolução, Instituto de Ciências Biológicas, Universidade Federal de Minas Gerais, Belo Horizonte, Brazil; ^4^National Institutes of Health, National Institute on Alcohol Abuse and Alcoholism, Rockville, MD, United States; ^5^Department of Psychiatry, Center for Alcohol Research in Epigenetics, University of Illinois, Chicago, IL, United States; ^6^Programa de Pos-Graduação em Epidemiologia, Universidade Federal de Pelotas, Pelotas, Brazil; ^7^Instituto de Pesquisa Rene Rachou, Fundação Oswaldo Cruz, Belo Horizonte, Brazil; ^8^Laboratory of Neuroimaging, National Institute on Alcohol Abuse and Alcoholism, National Institutes of Health, Bethesda, MD, United States

**Keywords:** alcohol dependence, CAGE, audit, LRRK2, polymorphisms, multiethnic

## Abstract

**Background:** Genetics influence the vulnerability to alcohol use disorders, and among the implicated genes, three previous studies have provided evidences for the involvement of *LRRK2* in alcohol dependence (AD). *LRRK2* expression is broadly dysregulated in postmortem brain from AD humans, as well as in the brain of mice with alcohol dependent-like behaviors and in a zebrafish model of alcohol preference. The aim of the present study was to evaluate the association of variants in the *LRRK2* gene with AD in multiethnic populations from South and North America.

**Methods:** Alcohol-screening questionnaires [such as CAGE and Alcohol Use Disorders Identification Test (AUDIT)] were used to determine individual risk of AD. Multivariate logistic regression analyses were done in three independent populations (898 individuals from Bambuí, Brazil; 3,015 individuals from Pelotas, Brazil; and 1,316 from the United States). Linkage disequilibrium and conditional analyses, as well as *in silico* functional analyses, were also conducted.

**Results:** Four *LRRK2* variants were significantly associated with AD in our discovery cohort (Bambuí): rs4768231, rs4767971, rs7307310, and rs1465527. Two of these variants (rs4768231 and rs4767971) were replicated in both Pelotas and US cohorts. The consistent association signal (at the *LRRK2* locus) found in populations with different genetic backgrounds reinforces the relevance of our findings.

**Conclusion:** Taken together, these results support the notion that genetic variants in the *LRRK2* locus are risk factors for AD in humans.

## Introduction

Alcohol use disorder (AUD) represents a global public health problem with profound personal, social, and economic costs (1). According to the Global Status Report on Alcohol and Health 2018 (2), from the World Health Organization (WHO), more than half of the world's population (57% or 3.1 billion people) consumed alcohol in the previous year. In 2016, the misuse of alcohol caused ~3 million deaths (5.3% of all deaths worldwide) (2). In Brazil, about 59% of the population consumes alcohol excessively, of whom ~14% are dependent on this drug (3). In the United States, 70.1% of the population above 18 years old consumed alcohol in 2017, a 4.4% increase compared with that in 2016 (4). The *Diagnostic and Statistical Manual of Mental Disorders* (DSM) and the *International Classification of Diseases* (ICD), characterize AUD by the continued use of alcohol despite negative psychological, biological, behavioral, and social consequences (5). The most recent version of DSM (DSM V) classifies the severity of AUD into mild, moderate, or severe, based on the number of symptoms present, with moderate and severe AUD largely equivalent to the DSM-IV diagnosis of alcohol dependence (6, 7).

There are several alcohol-screening questionnaires of which the Alcohol Use Disorders Identification Test (AUDIT) (8) and the CAGE (an acronym for the four questions present in this questionnaire: *Cut-down, Annoyed, Guilty*, and *Eye-opener*) (9) are among the most widely used in clinical and epidemiological research (10). AUD is influenced by multiple factors, including socio-environmental, developmental, physiological, and genetic factors (5, 11). From a genetic perspective, AUD has been proposed as the cumulative effects of multiple genes and their interactions with environmental factors, resulting in heterogeneous phenotypes (12). The genetic architecture of AUD is not yet fully understood, with the genes identified so far explaining a moderate proportion of the heritability attributed to this condition (varying between 50 and 60%) (13, 14). The most common variants associated with AUD are from genes involved in alcohol's metabolism including the genes for alcohol dehydrogenases (*ADH1B* and *ADH1C*) and aldehyde dehydrogenases (*ALDH2*) (15). In addition, genes related to the reward system have also been associated with AUD, including gene variants from monoaminergic and GABAergic systems (*DRD1, DRD2, DRD4, COMT, DAT1*, and *GABAA*) (12, 16, 17). Remarkably, most of these studies were conducted in European populations (18–20).

In a previous study, we found that the *Lrrk2* gene was upregulated specifically in heavy-drinking mice that simultaneously showed loss of control over their alcohol intake, but not in light drinking or non-compulsive mice (21). A transcriptional modulation of the *Lrrk2* gene was also observed in the brain of zebrafish with alcohol preference in which treatment with a selective inhibitor of LRRK2 reduced their preference for ethanol (22). Remarkably, it was recently demonstrated that *LRRK2* expression is dysregulated in the prefrontal cortex and nucleus accumbens of postmortem brain from AUD subjects (23). These results suggest a role for the LRRK2 pathway in compulsive alcohol intake. LRRK2 is a multifunctional protein with kinase and GTPase activities (24), involved in neuronal vesicle trafficking (25) and synaptic plasticity (26). In this regard, LRRK2 regulates the subcellular distribution of protein kinase A (PKA) and the phosphorylation of its targets, thus influencing glutamatergic neurotransmission (27). LRRK2 also controls dopamine D1 (DRD1) and D2 (DRD2) receptor trafficking, which are directly involved in dopaminergic neurotransmission (28). In humans, *LRRK2* variants are associated with familial and sporadic Parkinson's disease (PD) (29, 30).

Considering the convergent results found for the *LRRK2* gene from animal models and human brain, we hypothesized that *LRRK2* variants could represent genetic risk factors for alcohol dependence in humans. In the present study, we investigate the association of *LRRK2* variants and alcohol dependence in three distinct multiethnic cohorts, two from Brazil and one from the United States, and consistently show that intronic single-nucleotide polymorphisms (SNPs) were associated with alcohol dependence.

## Results

### Four Variants in *LRRK2* Are Associated With Alcohol Dependence in a Discovery Cohort From Bambuí, Brazil

In the first phase of this study, we tested 119 SNPs (covering the entire *LRRK2* gene, in addition to 10 kb of its 5′ and 3′ flanking regions) in 898 individuals from Bambuí, Brazil (Table 1). These numbers represent the dataset after stringent quality control (QC) to remove low-quality samples and SNPs. Phenotyping was performed using a Portuguese-adapted CAGE questionnaire. Seven hundred ninety-eight subjects were classified as controls (i.e., low/moderate risk of alcohol dependence) and 100 as cases (i.e., high risk of alcohol dependence). The median age (years) was 68 and 67 among controls and cases, respectively (not significant), and 22% of controls were male vs. 33% of cases (*P* < 0.05).

**Table 1:** Characteristics of the studied populations (after quality control).

	**Brazil, Bambu**í **(CAGE)**		**Brazil, Pelotas (AUDIT)**		**United States, NIH-NIAAA (AUDIT)**			
	**Control**	**Case**	**Control**	**Case**	**Control**		**Case**	
**Sample characteristics**								
Number of individuals	798	100	2,792	186	872		444	
Sex, male/female (%)	174 (22)/624 (78)	33 (33)/67 (67)	1,280 (46)/1,512(54)	139 (75)/47 (25)	487 (56)/385 (44)		325 (73)/119 (27)	
Median age, years (IQR)	68 (11)	67 (9)	30 (0)	30 (0)	30 (20)		46 (19)	
**Population genetic structure**								
PC1, mean (SD)^a^	0.0014 (0.41)	−0.011 (0.03)	−0.00099 (0.029)	−0.00099 (0.029)				
**Ancestry, mean (SD)%**^**b**^					**EUR**	**AFR**	**EUR**	**AFR**
					0.34 (0.39)	0.59 (0.38)	0.47 (0.38)	0.48 (0.38)

*Control, low/moderate risk of ethanol dependence; Case, high risk of ethanol dependence; SD, standard deviation; CAGE, alcohol dependence screening questionnaire; AUDIT, Alcohol Use Disorders Identification Test questionnaire; IQR, interquartile range; NIH-NIAAA, National Institutes of Health–National Institute on Alcohol Abuse and Alcoholism; IQR, interquartile range*.

a *Principal component 1 (PC1) from the principal component analysis (PCA)*.

b *Ancestry informative marker (AIM) scores for EUR, Europe; or AFR, Africa*.

**Figure 1:**
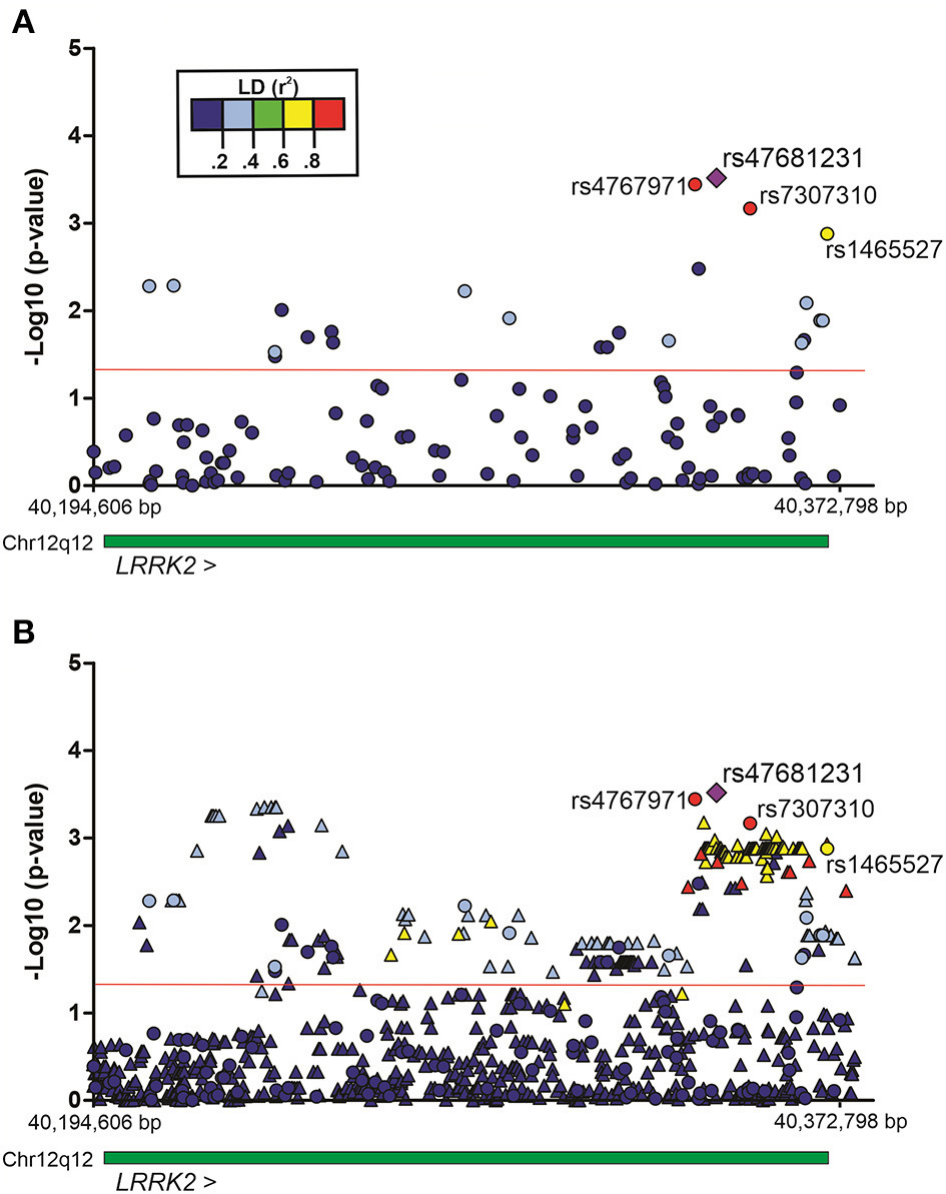
Regional plots of multivariate logistic regression results on alcohol dependence in the population of Bambuí, Brazil. Analyses were conducted under an additive genetic model, with sex, age, and the first principal component as covariates. **(A)** Genotyped variants in the *LRRK2* region. **(B)** Genotyped and imputed variants in the same region. Results [–Log_10_ (*P*-value)] for each variant are represented by circles (genotyped) or triangles (imputed). The purple diamond represents the top single-nucleotide polymorphism (SNP) rs47681231. The four SNPs highlighted in the figure were associated with alcohol dependence (after adjustment by false discovery rate, Benjamini–Hochberg). The green bar represents the extent of the *LRRK2* gene. The red line indicates *P* = 0.05. Region view: 12:40,194,606–40,372,798 (RefSeq: GRCh38). Linkage disequilibrium (LD, r^2^).

The association of SNPs with alcohol dependence was investigated by multivariate logistic regression (additive model), including sex and age as covariates. Additionally, the first principal component (PC1) derived from principal components analysis (PCA) was also included in the logistic model in order to account for population genetic structure. This initial screening stage revealed several SNPs that were nominally associated with alcohol dependence (*P* < 0.05) (Figure 1A). Supplementary Table 1 shows the detailed results for all SNPs evaluated in Bambuí. After results by false discovery rate (FDR; Benjamini–Hochberg) were adjusted, only four variants remained significant [rs4768231 (top SNP): OR = 2.03, *P* = 3.0 × 10^−4^, P_FDR_ = 0.021; rs4767971: OR = 2.00, *P* = 3.6 × 10^−4^, P_FDR_ = 0.021; rs7307310: OR = 1.94, *P* = 6.8 × 10^−4^, P_FDR_ = 0.027; rs1465527: OR = 1.82, *P* = 1.3 × 10^−3^, P_FDR_ = 0.039] (Table 2).

**Table 2:** Markers in the *LRRK2* locus associated with alcohol dependence in humans.

**SNP**	**Coordinate^d^**	**A1**	**MAF**	**F_**control**_**	**F_**case**_**	**OR**	**95% CI**	***P***	**P_**FDR**_**
**Discovery phase**									
***Brazil, Bambu**í **(CAGE)**^**a**^*									
rs4767971	Chr12:40338230	C	0.14	0.13	0.23	2.00	1.37–2.94	3.6 × 10^−4^	**0.021**
rs4768231	Chr12:40343381	G	0.14	0.12	0.23	2.03	1.38–2.99	3.0 × 10^−4^	**0.021**
rs7307310	Chr12:40351379	T	0.14	0.13	0.23	1.94	1.32–2.85	6.8 × 10^−4^	**0.027**
rs1465527	Chr12:40369805	C	0.19	0.17	0.27	1.82	1.26–2.62	1.3 × 10^−3^	**0.039**
**SNP**	**A1**	**MAF**	**F**_**control**_	**F**_**case**_	**OR**	**95% CI**	***P***		
**Replication phase**									
**Brazil, Pelotas (AUDIT)**^**b**^									
rs4767971	C	0.13	0.12	0.16	1.36	1.01–1.85	**0.049**		
rs4768231	G	0.19	0.18	0.23	1.33	1.02–1.74	**0.039**		
rs7307310	T	0.12	0.12	0.16	1.44	1.06–1.96	**0.020**		
rs1465527	C	0.13	0.13	0.16	1.31	0.96–1.79	0.083		
**SNP**	**A1**	**MAF**	**F**_**control**_	**F**_**case**_	**OR**	**95% CI**	***P***		
***United States, NIH-NIAAA (AUDIT)***^**c**^									
rs4767971	C	0.17	0.16	0.19	1.25	1.01–1.54	**0.039**		
rs4768231	G	0.26	0.24	0.29	1.23	1.02–1.48	**0.026**		
rs7307310	T	0.15	0.14	0.16	1.10	0.88–1.38	0.41		
rs1465527	C	Not genotyped							

*The statistical significance established for the discovery phase was P_FDR_ < 0.05. The significance level applied in the replication studies was P (raw) < 0.05*.

*A1, reference allele; MAF, minor allele frequency; Fcontrol, low/moderate risk of ethanol dependence; F_case_, high risk of ethanol dependence; OR, odds ratio; 95% CI, 95% confidence interval; P, P-value (additive model); P_FDR_, false discovery rate (FDR)-adjusted P-value (Benjamini–Hochberg); CAGE, alcohol screening questionnaire; AUDIT, Alcohol Use Disorders Identification Test questionnaire; NIH-NIAAA, National Institutes of Health–National Institute on Alcohol Abuse and Alcoholism; SNP, single-nucleotide polymorphism*.

a *Multivariate logistic regression—covariates: sex, age, and principal component 1 (PC1)*.

b *Multivariate logistic regression—covariates: sex and principal component 1 (PC1)*.

c *Multivariate logistic regression—covariates: sex, age, and ancestry informative marker (AIM) scores for Europe and Africa*.

d *Human genome assembly: GRCh38. Bold P-values denote statistical significance*.

To evaluate the contribution of genetic variants that were not directly genotyped, we carried out imputation of genotypes in the *LRRK2* locus (window 12:40,186,744–40,379,285). Multiple imputed variants were found to be nominally associated with the investigated phenotype (*P* < 0.05) (Figure 1B). Supplementary Table 2 shows the detailed results for all imputed variants evaluated in the discovery cohort. Despite these interesting results, no imputed variant revealed a stronger association signal in comparison with that found for the top genotyped SNP rs4768231. In addition, most imputed variants associated with alcohol dependence were in moderate-to-high linkage disequilibrium (LD) (*r*^2^) with rs4768231. Based on this, further analyses were restricted to the four genotyped SNPs associated with alcohol dependence.

### Variants in *LRRK2* Associated With Alcohol Dependence in Two Other Independent Cohorts

We investigated the association of rs4768231, rs4767971, rs7307310, and rs1465527 with alcohol dependence in 2,978 individuals from Pelotas, Brazil. Alcohol dependence risk was determined using a Portuguese-adapted AUDIT questionnaire, which has specific questions about alcohol use in the past 12 months and can be used to predict risk for alcohol dependence. As shown in Table 1 [Brazil, Pelotas (AUDIT)], 2,792 individuals were categorized as controls (i.e., low/moderate risk of alcohol dependence) and 186 as cases (i.e., high risk of alcohol dependence). Males were 46% of the controls and 75% of cases (*P* < 0.05) and as a birth cohort; all individuals had the same age (30 years). As shown in Table 2 (replication phase), through multivariate logistic regression analysis (covariates: sex and PC1), three of the four SNPs tested were also associated with alcohol dependence in this replication cohort (rs4768231: OR = 1.33, *P* = 0.039; rs4767971: OR = 1.36, *P* = 0.049; rs7307310: OR = 1.44, *P* = 0.020). The rs1465527 SNP showed only a trend association (*P* = 0.083).

The analysis of *LRRK2* variants was extended to a second replication cohort, composed of 1,316 North Americans [National Institutes of Health–National Institute on Alcohol Abuse and Alcoholism (NIH-NIAAA)] (Table 1). Post genotyping QC criteria similar to those applied in the Brazilian studies were used (see details in *Materials and Methods*). Alcohol dependence screening was also conducted with the AUDIT, resulting in 872 controls and 444 cases. Controls were on average 16 years younger than cases (*P* < 0.05). Males were 56% of controls and 73% of cases (*P* < 0.05). In addition to the covariates sex and age, ancestry informative marker (AIM) scores for Europe and Africa were included in the logistic regression model to account for population structure. Only the genotypes of rs4767971, rs4768231, and rs7307310 were available for this cohort. As can be seen in Table 2 (replication phase), two of these SNPs were associated with alcohol dependence (rs4767971: OR = 1.25, *P* = 0.039; rs4768231: OR = 1.23, *P* = 0.026). In order to explore the admixed nature of the US cohort, individuals were stratified in groups with European AIM score above or below the median. First, we evaluated whether the frequencies of rs4767971, rs4768231, and rs7307310 diverged between the two groups. We found that the minor allele frequencies (MAFs) of these SNPs were significantly higher in the group of individuals with the lowest degrees of EUR ancestry (Supplementary Table 3). Interestingly, rs4767971 and rs4768231 were associated (*P* < 0.05) with alcohol dependence only in individuals with the lowest degrees of European ancestry. Nevertheless, *a posteriori* power analysis evidenced that the dataset of individuals with the highest degrees of European ancestry had limited power (<20%) to detect the associations of rs4767971 or rs4768231 if such associations exist. A random-effects meta-analysis on these two sets of association results confirmed that rs4767971 and rs4768231 reached the significance level (*P* < 0.05) assumed for our replication phase. Importantly, the *LRRK2* alleles associated with a high risk of alcohol dependence were the same in the cohorts from Brazil and the United States.

Next, we conducted a meta-analysis on the three independent samples, by applying a random-effects model that assumes inter-study variability (Supplementary Table 4). As stated above, only the genotypes of rs4767971, rs4768231, and rs7307310 were available for the three cohorts. We observed that only rs4767971 (OR = 1.44, *P* = 4.4 × 10^−3^) and rs4768231 (OR = 1.42, *P* = 6.2 × 10^−3^) reached the significance level assumed for this analysis (*P* < 0.017, i.e., 0.05/3 tests).

### *LRRK2* rs4767971 and rs4768231 Are in Linkage Disequilibrium in Brazil and US Cohorts

Next, we investigated whether rs4767971 and rs4768231, the variants that were associated with alcohol dependence in the three cohorts, captured a single signal or were independently associated with the trait under study. As shown in Supplementary Figure 1, depending on the population analyzed, the rs4767971 and rs4768231 variants are in moderate-to-high LD [Brazil, Bambuí (*r*^2^ = 92); Brazil, Pelotas (*r*^2^ = 64); United States, NIH (*r*^2^ = 59)]. Therefore, it is likely that both SNPs might be capturing the same functional signal. We tested this hypothesis by carrying out conditional tests on these two polymorphisms, and we found that they were associated with alcohol dependence in an interdependent way. In the three cohorts, when including the rs4768231 genotypes as covariate in the regression models, the significant association signals for rs4767971 were completely abrogated (Bambuí, *P* = 0.79; Pelotas, *P* = 0.58; United States, *P* = 0.54).

### *In silico* Functional Analysis of *LRRK2* SNPs Associated With Human Alcohol Dependence

**Figure 2:**
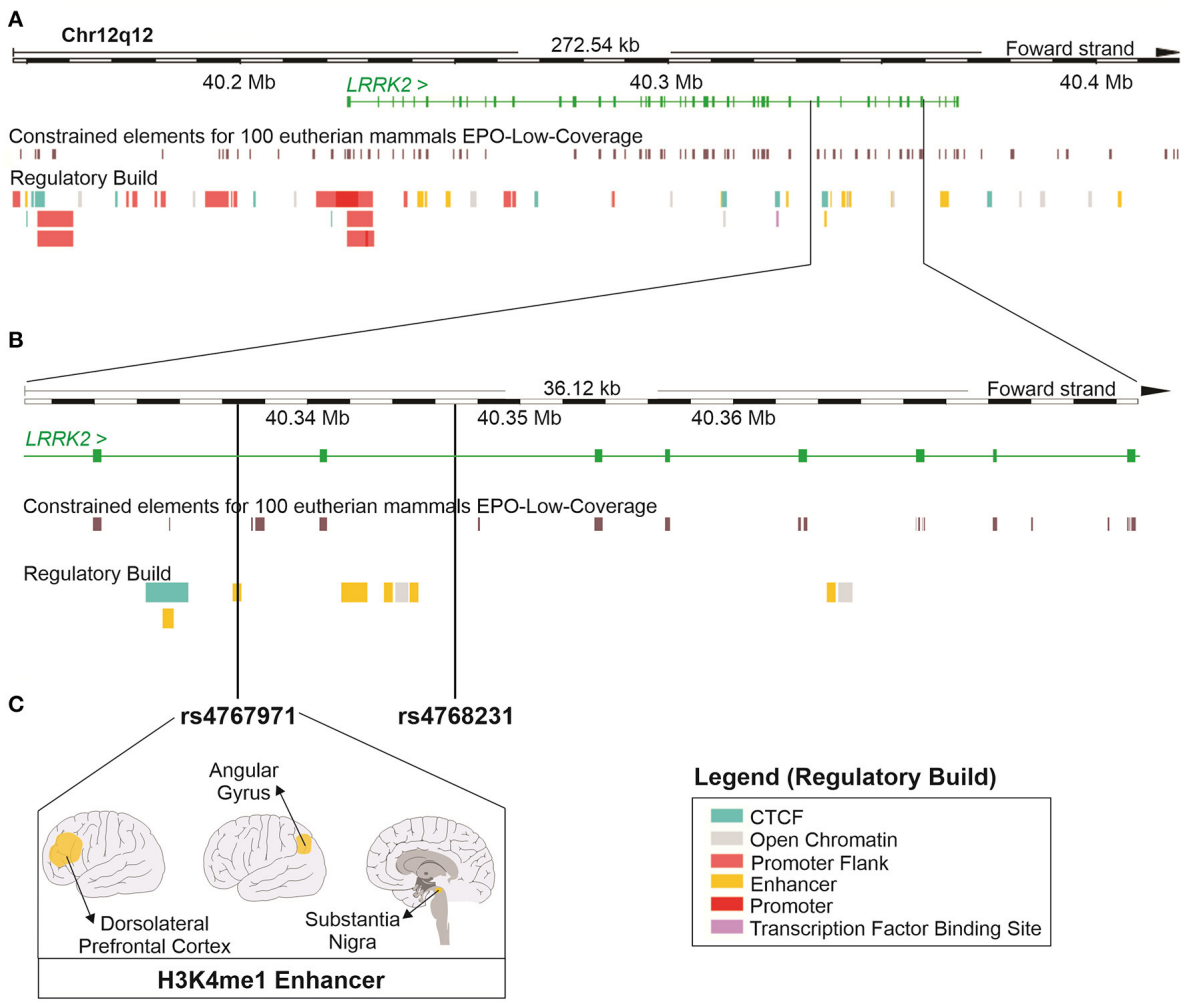
*In silico* functional study on the properties of the *LRRK2* variants associated with alcohol dependence in the three cohorts. **(A)** Schematic representation of the locus containing the *LRRK2* gene (12:40,146,744–40,419,285; RefSeq: GRCh38). The green continuous lines and rectangles represent introns and exons of the *LRRK2* gene, respectively. This region was cross-referenced with DNA sequence annotations, including the location of constrained elements for 100 eutherian mammals and the position of regulatory elements (Regulatory Build). **(B)** Magnified view of a region within the *LRRK2* gene where single-nucleotide polymorphisms (SNPs) rs4767971 and rs4768231 can be found. **(C)** rs4767971 is located within an H3K4me1 enhancer element identified in tissues from the brain dorsolateral prefrontal cortex, angular gyrus, and substantia nigra. Image created using the Ensembl Genome Browser (http://www.ensembl.org). H3K4me1: mono-methylation at the fourth lysine residue of the histone H3 protein.

To investigate the regulatory potential of rs4768231 and rs4767971, the *LRRK2* locus (12:40,146,744–40,419,285) was cross-referenced with genomic and epigenomic annotations, obtained from the Ensembl Genome Browser. As shown in Figure 2A, the *LRRK2* region was evaluated in terms of sequence constraint (across 100 eutherian mammals), chromatin segmentation states (evidence of promoter and enhancer marks), binding sites for transcription factors, and enrichment for marks of open chromatin. Figure 2B shows that both rs4768231 and rs4767971 are located in intronic sequences of *LRRK2*. This analysis also revealed that rs4767971 might have relevant functional consequences since it is located within an H3K4me1 element, which is a histone modification enriched at active and primed enhancers. The Roadmap Epigenomics Consortium (31) identified this regulatory element in only three brain regions [dorsolateral prefrontal cortex (dlPFC), angular gyrus, and substantia nigra] (Figure 2C). Collectively, these data support the biological plausibility of our findings.

## Discussion

Previous studies conducted by our group have shown altered expressions of *Lrrk2* in the striatum of mice with alcohol dependent-like behaviors (21) and in a zebrafish model of alcohol preference (22). Remarkably, in humans, the expression of *LRRK2* was found to be dysregulated in the prefrontal cortex and nucleus accumbens of postmortem brain from AUD subjects (23). In the present study, we first identified four variants (rs4768231, rs4767971, rs7307310, and rs1465527) in the *LRRK2* gene that were associated with alcohol dependence in a cohort from Brazil (Bambuí). We then investigated if these findings replicated in two independent cohorts. In both the Brazilian (Pelotas) and United States (NIH) replication cohorts, we showed that three (rs4768231, rs4767971, and rs7307310) and two (rs4768231 rs4767971) of these SNPs, respectively, were also associated with alcohol dependence. Consistently, the variants rs4768231 and rs4767971 are associated in an interdependent way (as suggested by the conditional analysis) with alcohol dependence in three distinct populations. It is worth noting that the odds ratios in the discovery and replication samples were in the same direction, indicating an increasing risk for alcohol dependence. This consistent effect found in populations with different genetic backgrounds suggests functional relevance for these SNPs or strong linkage to a causal variant yet to be identified. Furthermore, these replications provide evidences that our results are robust and rule out the possibility of spurious associations due to statistical/methodological artifacts. Taken together, these results support the notion that *LRRK2* variants are risk factors for alcohol dependence in humans.

The use of questionnaires to detect alcohol dependence is common in clinical routines and in epidemiologic studies (32, 33). The cohorts in this study were originally designed to address specific questions; this explains the difference in prevalence of cases. Bambuí and Pelotas are population-based cohorts, and alcohol dependence prevalence is 11 and 6%, respectively, while the alcohol dependence in Americas is 4.1% (2). On the other hand, the US NIH-NIAAA cohort, which is focused on AUD, has a selected population with high prevalence of alcohol dependence (34%). The cohort design also explains why we used different screening instruments for alcohol dependence (CAGE and AUDIT). The CAGE questionnaire, which was used in our discovery sample (Bambuí cohort), detects problems with alcohol consumption and dependence at any point in life (9). The AUDIT, which was used in the two replication cohorts (Pelotas and United States), detects current problems with alcohol consumption and dependence (34). Studies that have compared these two instruments show similar high specificity (90%) and sensitivity (80%) for screening alcohol dependence (34, 35). The CAGE was used in the Bambuí cohort, which comprised older individuals than in the other two cohorts (Table 1), for it shows better validity in the elderly than the AUDIT (35, 36), which screens for current problems (8). Nevertheless, both questionnaires are validated tools and show high correspondence for detecting alcohol dependence. De Moor and colleagues (37) showed in a sample of 5,870 twins and siblings and 4,420 additional family members that AUDIT and CAGE clustered on two highly correlated (0.74) underlying factors, thus suggesting that the items of the AUDIT and CAGE to a large extent represent one underlying problem drinking construct.

LD analysis in our multiethnic cohorts shows moderate-to-high correlation between rs4768231 and rs4767971 (${\text{r}}_{\text{Bambuí}}^{2}$ = 0.92; ${\text{r}}_{\text{Pelotas}}^{2}$ = 0.64; ${\text{r}}_{\text{United States}}^{2}$ = 0.59). For general comparison, reference populations from the four major continents (38) show different levels of LD for this pair of SNPs. Strong correlations are observed in Europeans (*r*^2^ = 0.80) and Asians (*r*^2^ = 0.85). On the other hand, Africans (*r*^2^ = 0.42) and Latin Americans (*r*^2^ = 0.34) show lower levels of LD between rs4768231 and rs4767971. These results can be explained by population-specific genetic architectures at the *LRRK2* locus. Even though genetic ancestry at the locus-segment level needs to be investigated, global ancestry in the Bambuí and Pelotas cohorts was largely European (78.5 and 76.1%, respectively) and to a lesser extent African (14.7 and 15.9%, respectively) (39). Interestingly, the *LRRK2* variants were associated with alcohol dependence only in individuals with European AIM score below the median in the US cohort.

Several genetic variants have been associated with alcohol abuse or dependence in humans (15, 40–43). The most commonly associated variants are in genes related to alcohol metabolism, as alcohol dehydrogenase family (*ADH1B, ADH1C*, and *ALDH2*) (43–46), and those related to reward pathways, as the *GABRA2* (40), and *DRD2* (43, 47). Besides that, other gene variants associated with alcohol dependence include *UTP20, ARL15, SLC22A18, PHLDA2, NAP1L4, SNORA54, CARS*, and *OSBPL5* (48). *JCAD, KLB*, and *GCKR* have also been associated with alcohol dependence as assessed with the AUDIT (43). Most of these studies were conducted in European-derived populations (18–20). More recent analyses of non-European populations have suggested the existence of additional variants associated with AUD (44, 45, 49), emphasizing the importance of considering diverse populations in genetic studies in order to obtain a more complete understanding of the underpinnings of this complex condition. To the best of our knowledge, our study (involving multiethnic populations from South and North America) is the first to demonstrate that LRRK2 variants are associated with alcohol dependence in humans. Specifically, no genetic variation in LRRK2 has been found to reach genome-wide significance level in recently published and well-powered genome-wide association studies (GWASs) on alcohol dependence (44, 46, 50). These apparently discordant results can be explained by the intrinsic characteristics of the investigated populations, different study designs, and coverage of the used SNP chips.

Previous studies have shown an association of *LRRK2* SNP variants with familial and sporadic PD (29, 30). The most prevalent *LRRK2* mutation in PD causes an amino acid substitution in the position 2,019 (G2019S) of the protein, which increases the catalytic kinase activity of LRRK2 (51). To verify if PD and alcohol dependence are genetically correlated, we accessed the GWAS catalog (https://www.ebi.ac.uk/gwas/home), a curated collection of human GWASs. We identified at least two other risk loci, at *IGSF9B* and *SLC39A8*, shared by the two conditions (44, 52–54). More recently, SNPs in *LRRK2* were associated with cancer (55, 56) and inflammatory conditions (including infectious and autoimmune diseases) (57). LRRK2 is a complex protein with different functional regions, including protein–protein interaction, GTPase, and kinase activity domains (24). Alterations in LRRK2 kinase function have been shown to affect dopaminergic function (26, 28, 58, 59) via disruption of synaptic vesicle formation and trafficking (60), signal transmission (26), and receptor function (28). Inhibition of LRRK2 in mice striatum increased the mobilization and recycling of synaptic vesicles and improved dopamine release (58). Using the inhibitor GNE-0877 of LRRK2 kinase activity, our group observed a significant reduction of alcohol preference in the zebrafish model (22).

According to chromatin immunoprecipitation assay (ChIP) (31), the variant rs4767971 is located in an enhancer region marked by a histone methylation, H3k4me1, only in three brain regions (substantia nigra, angular gyrus, and dlPFC). These enhancer regions are methylated or acetylated according to their activity status (poised, primed, or active) (61). H3K4me1 is a marker of primed or poised enhancer and is responsible for the fine-tune of transcriptional regulation in response to environmental modifications (62). Loss of H3K4me1 marker might affect gene expression partially. There is no information in public databases, such as the GTEx portal (63), about the effect of rs4767971 on *LRRK2* expression in brain tissues. Therefore, more studies are necessary to understand how this variant influences gene expression.

In conclusion, this study demonstrated, for the first time, the association of *LRRK2* variants with alcohol dependence in three different human populations. Studies that combine diverse populations are highly relevant in order to determine how genes and different environmental factors can influence a particular phenotype and to examine the consistency of established associations across different populations. Further studies are required to evaluate the role of the variants associated with alcohol dependence in *LRRK2* expression.

## Materials and Methods

### Population Samples

#### Brazil, Bambuí

The Bambuí cohort (discovery cohort) was established in Bambuí City, in Minas Gerais State, in Southeast Brazil. The population eligible for the cohort study consisted of all residents who were 60 years or older on January 1997, identified after a complete census of the city. Of the 1,742 eligible residents (individuals ≥60 years), 1,606 were recruited and completed the CAGE questionnaire (9) to identify alcohol dependence. A total of 1,442 individuals were successfully genotyped as part of the EPIGEN initiative (64) (https://epigen.grude.ufmg.br). Further details of the Bambuí cohort can be seen in Lima-Costa et al. (65).

#### Brazil, Pelotas

The Pelotas cohort (replication 1 cohort) was established in Pelotas City. Throughout 1982, the births from the three maternity hospitals in the city, which account for 99.2% of all births, were recorded on a daily basis. The 5,914 live-born infants whose families lived in the urban area constituted the cohort. Further details on the Pelotas (1982) birth cohort can be seen in Horta et al. (66). At 30 years of age, 3,089 participants answered the WHO's AUDIT (8). Furthermore, 3,015 of these individuals were genotyped as part of the EPIGEN initiative. According to the EPIGEN-Brazil initiative's data usage policy, the Pelotas sample was only available for replication purposes.

### United States, National Institutes of Health–National Institute on Alcohol Abuse and Alcoholism

A total of 2,152 participants of African American or Caucasian ethnicity (including African/European admixed individuals) were selected from an existing NIH-NIAAA database for whom genotyping data that passed our standardized QC were available (see below). Those with self-reported American Indian or Alaska Native, Asian, Native Hawaiian, or Other Pacific Islander ethnicity (*n* = 101) were excluded. A total of 1,316 participants completed the AUDIT.

### Ethics Statement and Accordance With Guidelines and Regulations

Participants from the Brazilian cohorts provided written informed consent to participate in the study, which was approved by Brazil's National Research Ethics Committee (CONEP), as part of the EPIGEN-Brazil initiative (resolution number: 15895). Participants in the NIH cohort provided written informed consent to participate in the study, which was approved by the Institutional Review Board at NIH. All participants agreed to genotyping of their samples. All methods and protocols were performed in accordance with the principles of the Declaration of Helsinki.

### Definition of Alcohol Dependence and Phenotyping

Alcohol dependence in the Bambuí cohort was identified using the CAGE questionnaire. In this test, individuals answer “yes” or “no” to four questions: (1) Have you ever felt you needed to cut down on your drinking? (2) Have people annoyed you by criticizing your drinking? (3) Have you ever felt guilty about drinking? (4) Have you ever felt you needed a drink first thing in the morning to steady your nerves or to get rid of a hangover? These questions cover alcohol consumption during lifetime and are specific to dependence behavior. The Portuguese validation of the CAGE questionnaire was carried out by Masur and Monteiro (67), who estimated a sensitivity of 88% and a specificity of 83% to detect alcohol dependence. In the present study, following the recommendations from the seminal CAGE publication (68), individuals who answered affirmatively to two or more questions were categorized as cases (i.e., high risk of alcohol dependence). Individuals responding “no” to all questions were classified as controls (i.e., low/moderate risk of alcohol dependence). The 539 individuals who reported no alcohol consumption or answered yes to only one question were excluded.

Phenotyping of the Pelotas and the US cohorts was done using the AUDIT, widely used to detect high-risk drinking with three questions on alcohol consumption (consumption score), three questions on drinking behavior and dependence (dependence score), and four other questions on problems related to drinking (alcohol-related problems score). The AUDIT questionnaire was found to have 92% sensitivity and 94% specificity to detect alcohol dependence (8). Individual risk level for alcohol dependence was inferred by the combination of results obtained from the total score (maximum score possible = 40) and the dependence score (maximum score possible = 12). The thresholds for the AUDIT tool were defined as recommended by the AUDIT Decision Tree (https://auditscreen.org) and by user manuals provided by several health committees, such as the UCLA Medical Staff Health Committee (69). Individuals with an AUDIT total score higher than 15 and a dependence score of 4 or more were classified as cases (i.e., high risk of alcohol dependence), and those with AUDIT total score of 15 or less and dependence score below 4 were included in the control group (i.e., low/moderate risk of alcohol dependence). Both the AUDIT and CAGE have been extensively validated as screening questionnaires for alcohol dependence.

### Single-Nucleotide Polymorphism Genotyping and Quality Control

Individuals from the Brazilian cohorts were genotyped as part of the EPIGEN-Brazil initiative using the Illumina HumanOmni 2.5–8v1 BeadChip panel (Illumina, San Diego, CA). Genotyping for the US-NIH cohort was performed using the Illumina Human OmniExpress Exomearray (Illumina, San Diego, CA).

Standardized QC was performed to exclude individuals presenting with the following: (i) inconsistency between registered and genetic sex, based on X-chromosome markers, using PLINK v1.9 (70) (–check-sex); and (ii) close relationship estimated by kinship coefficients for each pair of individuals, using a method implemented in the REAP software (Relatedness Estimation in Admixed Populations) (71). Pairs of individuals were considered closely related if the estimated kinship coefficient between them was ≥0.1; and (iii) more than 1% of undetermined genotypes, using PLINK v1.9 (–mind 0.01). After sample QC, five individuals were excluded from the Bambuí cohort, and the other 37 were excluded from the Pelotas cohort. QC was also performed to eliminate SNPs showing (i) significant deviation from the Hardy–Weinberg equilibrium [*P* < 10^−5^ (–hwe 0.00001), based on controls only]; (ii) more than 1% of undetermined genotypes (–geno 0.01); and (iii) MAF <1% (–maf 0.01). All stages of SNP QC were also carried out using PLINK v1.9.

For the first phase of this study, after genomic QC, 119 SNPs in the *LRRK2* region (including 10 kb of its 5′ and 3′ flanks; 12:40,186,744–40,379,285; RefSeq: GRCh38) were identified and analyzed in 898 individuals from Bambuí. All the SNPs explored in the replication phases passed the genomic QC and were analyzed in 2,978 individuals from Pelotas and in 1,316 individuals from the United States.

### Genotype Imputation

The procedures for genotype imputation in the Bambuí cohort were described by Magalhães and colleagues (72). Briefly, imputation was based on the EPIGEN-5M+1KGP reference panel, which is a mergence of the 1000 Genomes Project Phase 3 haplotypes panel, version 20130502 (73), and our unpublished EPIGEN-5M panel, which comprises 4,102,271 SNPs for 265 Brazilians. SHAPEIT2 (74) was used to check the consistency of the SNP's strand on the target data and the reference panels, and PLINK v1.9 software was used to flip the strands in cases of inconsistency (–flip). Target dataset was phased using the EPIGEN-5M dataset as phasing reference. Genotype imputation was performed by IMPUTE2 v2.3.2 (75). The IMPUTE2 *info score* was used as a metric of imputation quality. Only imputed variants with *info score* ≥0.7 and MAF ≥1% were considered for analysis. In this context, imputed genotypes of 685 variants in the *LRRK2* region (12:40,186,744–40,379,285; RefSeq: GRCh38) were evaluated.

### Linkage Disequilibrium and Population Genetic Structure

LD (*r*^2^) analysis was performed using HAPLOVIEW v4.2 (76).

To explore the admixed nature of the Brazilian samples, we conducted PCA (77), using PLINK v1.9. Remarkably, only PC1 accounted for more than 5% of data variance in both Brazilian samples [Bambuí (PC1 = 22.2%) and Pelotas (PC1 = 39.2%)] (Supplementary Figure 2). Thus, only this more informative PC was used to adjust for population stratification.

Ethnic origin for individual study subjects from the NIH cohort was characterized using a panel of 2,500 AIMs and individual comparison with the 51 worldwide populations represented in the Human Genome Diversity Cell Line Panel of the Human Genome Diversity Project (HGDP) and Center d'Etude du Polymorphisme Humain (CEPH), which includes 1,051 individuals (http://www.cephb.fr/HGDP-CEPH-Panel). Ancestry scores were calculated using Structure, version 2.2 (http://pritch.bsd.uchicago.edu/structure.html), where data for the CEPH diversity panel was run along with data for a single study subject (78, 79). The number of ethnic clusters (K) was defined by running the data with different K values and computing the probability of K = n. The six-factor solution was optimal for this marker set and closely replicates solutions found by Rosenberg et al. (80), wherein all the non-Arabic African populations in the Human Genome Diversity Cell Line Panel are identified by a single African factor in this six-factor solution (Africa, Europe, Asia, Far East Asia, Oceania, and America). In this dataset of African American/Black and European ancestry, we specifically focused on the African and European AIM scores.

### *In silico* Functional Analysis

Comparative genomic and epigenomic data for the *LRRK2* locus (12:40,146,744–40,419,285; RefSeq: GRCh38) were obtained from the Ensembl Genome Browser (http://www.ensembl.org). The positions of SNPs associated with alcohol dependence were cross-referenced with several sequence annotations, including (i) *LRRK2* coordinates (positions of introns and exons); (ii) genomic evolutionary rate profiling-constrained elements for 100 eutherian mammals (GERP conservation scores) (81); (iii) presence of consensus sequences for transcription factors (82); (iv) chromatin accessibility (DNase I hypersensitive sites); and (v) chromatin segmentation states (histone marks for promoter, promoter flank, enhancer, and CTCF). These last two types of information were obtained from large epigenomic consortia, such as the ENCODE Project Consortium (83) and the Roadmap Epigenomics Consortium (31).

### Statistical Analysis

Frequencies of *LRRK2* variants were compared between controls and cases, under an additive genetic model. The effective number of independent marker loci in the Bambuí discovery sample (Meff = 290) was estimated by the Single Nucleotide Polymorphism Spectral Decomposition (SNPSpD) software (84). Statistical power was estimated using the Quanto software. This factor depends on the effect of each polymorphism [allele frequencies and associated relative risks (OR)], the size of the sample, and the degree of type I error. Using an additive genetic model and the experiment-wide significance threshold required to keep type I error rate at 5% (1.7 × 10^−4^), the Bambuí cohort has ≥70% power to describe a polymorphism with a frequency equal to 19%, which determines a relative risk of 1.8.

In the Bambuí sample, sex, age, and PC1 (to account for population genetic structure) were included as covariates in the logistic regression model. Since all individuals had the same age (30 years) in the Pelotas cohort, only sex and PC1 were used as covariates. In the US cohort, sex, age, and AIM scores for Europe and Africa (also to correct for eventual population stratification) were integrated in the logistic regression analysis. Results are described as estimates of OR and confidence interval (CI). In the discovery phase (Bambuí), a FDR (Benjamini–Hochberg) adjustment was applied to limit the probability of false-positive results. After that, P_FDR_ < 0.05 was taken as significant. In the replication phase (Pelotas and United States), the significance level was *P* (raw) < 0.05. To avoid unnecessary increase in the burden of multiple testing in the Bambuí discovery analysis, genotyped and imputed SNPs were evaluated in different moments. To combine association results, we carried out random-effects meta-analysis (assuming inter-study variability). All these analyses were conducted using PLINK v1.9 software.

## Data Availability Statement

The EPIGEN data are deposited in the European Nucleotide Archive [PRJEB9080 (ERP010139) Genomic Epidemiology of Complex Diseases in Population-Based Brazilian Cohorts], Accession No. EGAS00001001245, under EPIGEN Committee Controlled Access mode. We used de-identified genotype and phenotype data from NIAAA protocols 14-AA-0181, 98-AA-009, and 05-AA-0121, which are not accessible online.

## Ethics Statement

The studies involving human participants were reviewed and approved by Brazil's National Research Ethics Committee (CONEP) And Institutional Review Board at NIH. The patients/participants provided their written informed consent to participate in this study.

## Author Contributions

PRSO, LOM, and ALBG conceived the project. PRSO, LOM, NMA, HPS, AD, and ALBG performed the association and functional analysis. PRSO, LOM, DASS, LMC, BLH, MFL-C, MLB, CEW, NDV, and ALBG participated in the data collection and interpretation of results. All authors contributed to the writing and editing of the manuscript.

## Conflict of Interest

The authors declare that the research was conducted in the absence of any commercial or financial relationships that could be construed as a potential conflict of interest.
