# Supplementary material for: LRRK2 Gene Variants Associated With a Higher Risk for Alcohol Dependence in Multiethnic Populations
Source: Front Psychiatry. 2021 May 31;12:665257. doi: 10.3389/fpsyt.2021.665257 (PMC8202767; doi:10.3389/fpsyt.2021.665257)
Supplement: Supplementary file 1 [file Data_Sheet_1.doc]

***LRRK2* gene variants associated with a higher risk for alcohol dependence in multiethnic populations**

Pablo Rafael Silveira Oliveira, Lorena Oliveira de Matos, Nathalia Matta Araujo, Hanaísa P. Sant Anna, Daniel Almeida da Silva e Silva, Andresa K. Andrade Damasceno, Luana M. de Carvalho, Bernardo L. Horta, Maria Fernanda Lima-Costa, Mauricio Lima Barreto, Corinde E. Wiers, Nora D. Volkow and Ana Lúcia Brunialti Godard

| **Content** |  |
| --- | --- |
| **Figure S1** | 3 |
| **Figure S2** | 4 |
| **Table S1** | 5 |
| **Table S2** | 8 |
| **Table S3** | 24 |
| **Table S4** | 25 |


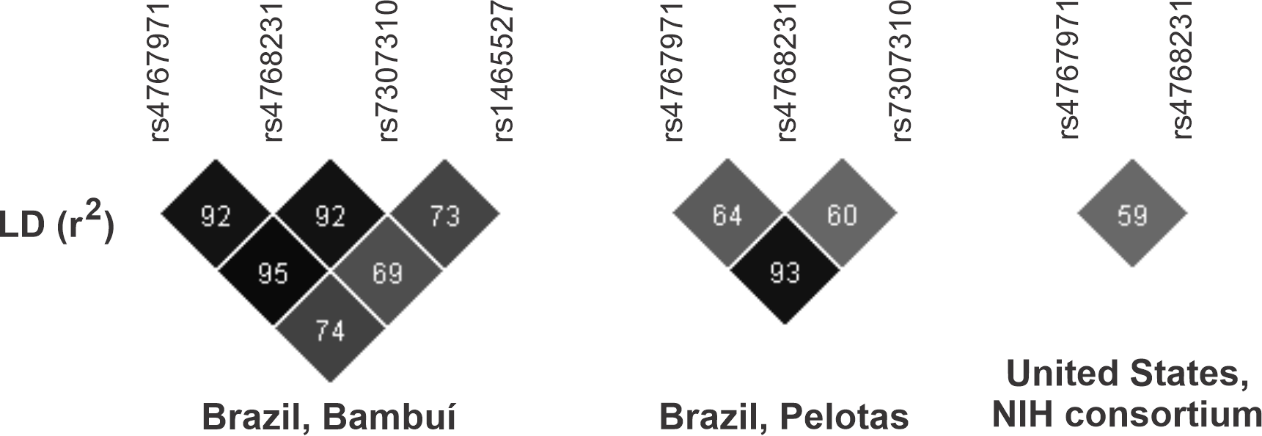


**Figure S1. Linkage disequilibrium (LD) of SNPs associated with alcohol dependence in the studied populations.** LD (r^2^) between pairs of SNPs were estimated using HAPLOVIEW v4.2.


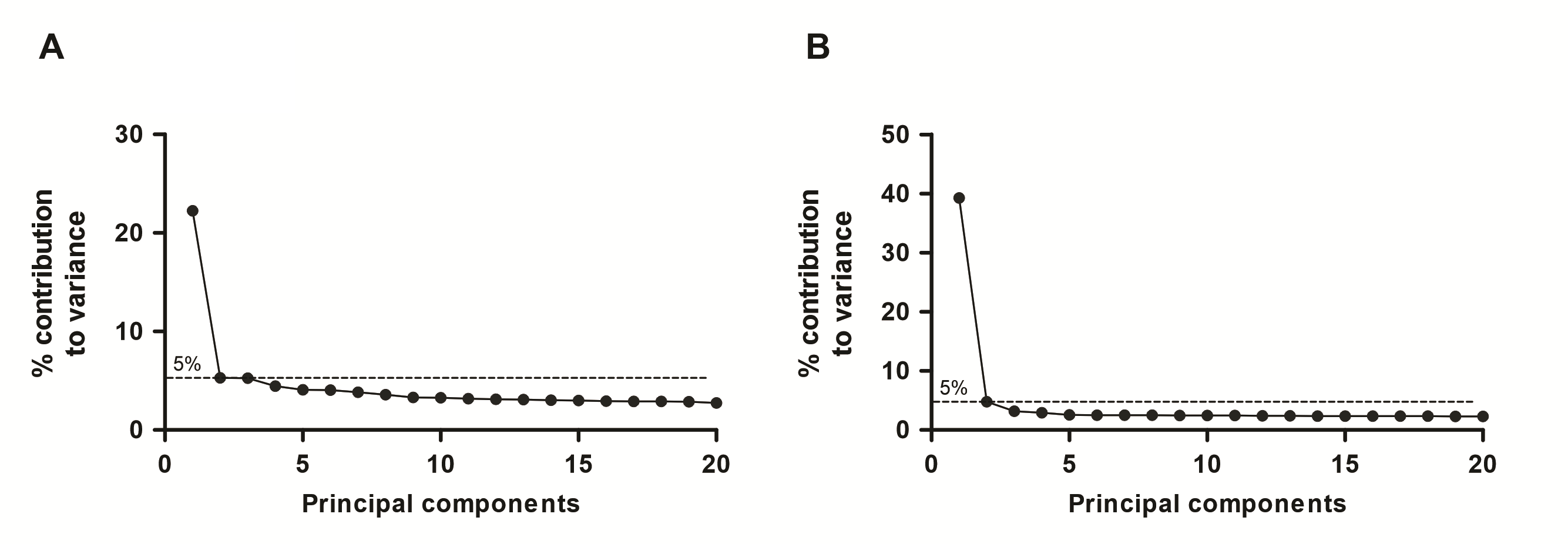


**Figure S2. Principal components analysis (PCA) of the Bambuí and Pelotas samples.** Scree plots showing the % of contribution to data variance for the first 20 principal components (PCs) in Bambuí (A) and Pelotas (B). In both cohorts, the eigenvalues for the first PC is the only greater than 5%.

| **Table S1. *LRRK2* locus: genotyped SNPs evaluated in the discovery cohort (Bambuí, Brazil)** | | | | | | | | | |
| --- | --- | --- | --- | --- | --- | --- | --- | --- | --- |
| **SNP** | **Coordinate (chr12)^a^** | **A1** | **MAF** | **F_control_** | **F_case_** | **OR** | **95% CI** | **P** | **P_FDR_** |
| rs10878208 | 40194606 | T | 0.295 | 0.29 | 0.32 | 1.16 | 0.82-1.62 | 0.41 | 0.73 |
| rs113553365 | 40195096 | A | 0.013 | 0.01 | 0.02 | 1.28 | 0.34-4.80 | 0.71 | 0.97 |
| rs2708418 | 40198423 | A | 0.391 | 0.39 | 0.41 | 1.08 | 0.79-1.48 | 0.63 | 0.93 |
| rs2708421 | 40199601 | T | 0.487 | 0.49 | 0.49 | 1.08 | 0.80-1.47 | 0.61 | 0.93 |
| rs1491935 | 40202365 | T | 0.299 | 0.30 | 0.33 | 1.21 | 0.86-1.71 | 0.27 | 0.56 |
| rs2723260 | 40207906 | T | 0.090 | 0.08 | 0.14 | 1.98 | 1.23-3.19 | 0.0052 | 0.089 |
| rs2723261 | 40208006 | C | 0.429 | 0.43 | 0.44 | 0.99 | 0.73-1.34 | 0.95 | 0.98 |
| rs11175593 | 40208138 | T | 0.028 | 0.03 | 0.03 | 0.93 | 0.34-2.54 | 0.89 | 0.97 |
| rs1491932 | 40208422 | A | 0.401 | 0.40 | 0.41 | 1.00 | 0.73-1.36 | 0.98 | 0.99 |
| rs6581593 | 40208963 | T | 0.065 | 0.07 | 0.04 | 0.59 | 0.28-1.26 | 0.17 | 0.46 |
| rs2279535 | 40209514 | T | 0.382 | 0.38 | 0.38 | 0.94 | 0.68-1.28 | 0.68 | 0.97 |
| rs1388593 | 40213764 | A | 0.090 | 0.08 | 0.14 | 1.98 | 1.23-3.19 | 0.0052 | 0.089 |
| rs10878220 | 40215078 | T | 0.379 | 0.38 | 0.33 | 0.81 | 0.59-1.12 | 0.20 | 0.49 |
| rs11613206 | 40215779 | A | 0.101 | 0.10 | 0.10 | 0.92 | 0.54-1.59 | 0.78 | 0.97 |
| rs2638272 | 40216081 | G | 0.421 | 0.42 | 0.43 | 0.99 | 0.73-1.34 | 0.92 | 0.97 |
| rs12827541 | 40216177 | T | 0.388 | 0.39 | 0.35 | 0.85 | 0.62-1.17 | 0.32 | 0.61 |
| rs10878222 | 40216916 | A | 0.379 | 0.39 | 0.33 | 0.81 | 0.59-1.12 | 0.20 | 0.49 |
| rs57374150 | 40218182 | G | 0.022 | 0.02 | 0.03 | 1.00 | 0.36-2.80 | 1.00 | 1.00 |
| rs76904798 | 40220632 | T | 0.124 | 0.13 | 0.10 | 0.74 | 0.44-1.22 | 0.23 | 0.53 |
| rs75024792 | 40221491 | C | 0.022 | 0.02 | 0.03 | 1.07 | 0.38-2.99 | 0.90 | 0.97 |
| rs116859512 | 40221574 | T | 0.022 | 0.02 | 0.02 | 0.64 | 0.19-2.20 | 0.48 | 0.79 |
| rs28365207 | 40222612 | G | 0.107 | 0.11 | 0.11 | 0.91 | 0.53-1.54 | 0.72 | 0.97 |
| rs12304648 | 40223419 | T | 0.012 | 0.01 | 0.02 | 0.94 | 0.25-3.44 | 0.92 | 0.97 |
| rs1388597 | 40224295 | C | 0.028 | 0.03 | 0.03 | 0.92 | 0.34-2.50 | 0.87 | 0.97 |
| rs2256408 | 40225280 | G | 0.011 | 0.01 | 0.02 | 1.48 | 0.41-5.39 | 0.55 | 0.88 |
| rs2256286 | 40225740 | A | 0.011 | 0.01 | 0.02 | 1.48 | 0.41-5.39 | 0.55 | 0.88 |
| rs17465751 | 40227079 | T | 0.037 | 0.04 | 0.03 | 0.66 | 0.25-1.74 | 0.40 | 0.73 |
| rs12230685 | 40228988 | T | 0.167 | 0.17 | 0.16 | 0.95 | 0.62-1.45 | 0.81 | 0.97 |
| rs11175655 | 40229925 | A | 0.098 | 0.10 | 0.08 | 0.68 | 0.38-1.20 | 0.19 | 0.48 |
| rs1352879 | 40232428 | A | 0.028 | 0.03 | 0.05 | 1.57 | 0.73-3.40 | 0.25 | 0.55 |
| rs2723270 | 40237925 | G | 0.031 | 0.03 | 0.06 | 2.35 | 1.09-5.05 | 0.029 | 0.15 |
| rs10878245 | 40237989 | T | 0.489 | 0.48 | 0.44 | 1.39 | 1.03-1.88 | 0.033 | 0.16 |
| rs10878247 | 40238298 | T | 0.305 | 0.30 | 0.32 | 1.05 | 0.76-1.45 | 0.76 | 0.97 |
| rs10878249 | 40239512 | C | 0.361 | 0.37 | 0.27 | 0.64 | 0.46-0.90 | 0.0098 | 0.11 |
| rs6581622 | 40240356 | C | 0.310 | 0.31 | 0.32 | 1.03 | 0.75-1.41 | 0.88 | 0.97 |
| rs11564153 | 40241189 | T | 0.030 | 0.03 | 0.03 | 0.83 | 0.31-2.26 | 0.72 | 0.97 |
| rs2404580 | 40245759 | G | 0.471 | 0.48 | 0.39 | 0.70 | 0.51-0.94 | 0.020 | 0.13 |
| rs10878258 | 40247890 | G | 0.267 | 0.27 | 0.27 | 1.02 | 0.72-1.44 | 0.91 | 0.97 |
| rs7955902 | 40251455 | A | 0.309 | 0.32 | 0.23 | 0.66 | 0.46-0.93 | 0.017 | 0.13 |
| rs1491938 | 40251828 | T | 0.476 | 0.49 | 0.39 | 0.71 | 0.52-0.95 | 0.023 | 0.13 |
| rs11564186 | 40252436 | A | 0.017 | 0.02 | 0.03 | 2.05 | 0.77-5.41 | 0.15 | 0.45 |
| rs1907633 | 40256549 | T | 0.141 | 0.14 | 0.13 | 0.85 | 0.54-1.33 | 0.48 | 0.79 |
| rs2723264 | 40258718 | T | 0.185 | 0.18 | 0.21 | 1.11 | 0.76-1.63 | 0.59 | 0.92 |
| rs17465870 | 40259911 | T | 0.139 | 0.14 | 0.10 | 0.71 | 0.43-1.17 | 0.18 | 0.48 |
| rs10878278 | 40260186 | T | 0.167 | 0.17 | 0.18 | 1.04 | 0.69-1.57 | 0.84 | 0.97 |
| rs11564183 | 40261905 | T | 0.102 | 0.10 | 0.10 | 0.87 | 0.51-1.50 | 0.62 | 0.93 |
| rs11175769 | 40262377 | G | 0.400 | 0.41 | 0.33 | 0.74 | 0.54-1.03 | 0.072 | 0.29 |
| rs4293189 | 40263423 | A | 0.398 | 0.41 | 0.33 | 0.75 | 0.54-1.03 | 0.078 | 0.29 |
| rs11564113 | 40264066 | C | 0.014 | 0.02 | 0.01 | 0.74 | 0.16-3.39 | 0.70 | 0.97 |
| rs11175780 | 40265294 | T | 0.116 | 0.11 | 0.13 | 1.03 | 0.66-1.61 | 0.89 | 0.97 |
| rs11175793 | 40268237 | A | 0.117 | 0.12 | 0.11 | 0.76 | 0.46-1.26 | 0.28 | 0.56 |
| rs10784470 | 40269794 | T | 0.246 | 0.25 | 0.20 | 0.81 | 0.55-1.18 | 0.27 | 0.56 |
| rs11564129 | 40276137 | C | 0.092 | 0.09 | 0.08 | 0.79 | 0.45-1.37 | 0.40 | 0.73 |
| rs10506151 | 40277196 | A | 0.117 | 0.12 | 0.11 | 0.93 | 0.57-1.52 | 0.77 | 0.97 |
| rs10878307 | 40278187 | G | 0.058 | 0.06 | 0.07 | 1.32 | 0.68-2.53 | 0.41 | 0.73 |
| rs11175847 | 40282398 | T | 0.386 | 0.40 | 0.31 | 0.73 | 0.52-1.02 | 0.062 | 0.27 |
| rs10784486 | 40283227 | A | 0.327 | 0.32 | 0.41 | 1.58 | 1.14-2.18 | 0.0059 | 0.089 |
| rs11175884 | 40288634 | A | 0.021 | 0.02 | 0.02 | 0.83 | 0.27-2.51 | 0.73 | 0.97 |
| rs76434788 | 40290890 | G | 0.026 | 0.02 | 0.04 | 1.90 | 0.78-4.64 | 0.16 | 0.45 |
| rs5006481 | 40293896 | C | 0.323 | 0.31 | 0.40 | 1.52 | 1.10-2.10 | 0.012 | 0.11 |
| rs7966550 | 40294893 | C | 0.122 | 0.12 | 0.12 | 0.96 | 0.61-1.54 | 0.88 | 0.97 |
| rs17443815 | 40296259 | G | 0.388 | 0.40 | 0.32 | 0.74 | 0.53-1.03 | 0.078 | 0.29 |
| rs79400217 | 40296715 | A | 0.032 | 0.03 | 0.02 | 0.55 | 0.19-1.63 | 0.28 | 0.56 |
| rs10878336 | 40299423 | C | 0.123 | 0.12 | 0.12 | 0.83 | 0.51-1.35 | 0.45 | 0.77 |
| rs10784498 | 40303632 | A | 0.438 | 0.44 | 0.40 | 0.76 | 0.55-1.05 | 0.095 | 0.34 |
| rs7133914 | 40309109 | A | 0.114 | 0.12 | 0.10 | 0.76 | 0.45-1.26 | 0.28 | 0.56 |
| rs11175964 | 40309185 | A | 0.090 | 0.09 | 0.08 | 0.70 | 0.38-1.27 | 0.24 | 0.53 |
| rs17443854 | 40310076 | T | 0.021 | 0.02 | 0.02 | 1.18 | 0.38-3.61 | 0.78 | 0.97 |
| rs11564128 | 40312033 | T | 0.158 | 0.16 | 0.12 | 0.70 | 0.44-1.10 | 0.12 | 0.39 |
| rs11175985 | 40313476 | T | 0.099 | 0.10 | 0.07 | 0.68 | 0.38-1.25 | 0.22 | 0.51 |
| rs4768227 | 40315697 | T | 0.450 | 0.44 | 0.47 | 1.43 | 1.04-1.95 | 0.026 | 0.14 |
| rs10748029 | 40317244 | C | 0.450 | 0.44 | 0.47 | 1.43 | 1.04-1.95 | 0.026 | 0.14 |
| rs11176013 | 40320071 | A | 0.450 | 0.44 | 0.46 | 1.46 | 1.07-2.00 | 0.018 | 0.13 |
| rs11564148 | 40320099 | A | 0.235 | 0.24 | 0.20 | 0.87 | 0.59-1.29 | 0.49 | 0.80 |
| rs4768230 | 40321500 | A | 0.236 | 0.24 | 0.20 | 0.86 | 0.58-1.26 | 0.44 | 0.76 |
| rs4423250 | 40321788 | T | 0.107 | 0.11 | 0.11 | 0.98 | 0.61-1.57 | 0.92 | 0.97 |
| rs10878372 | 40322892 | G | 0.196 | 0.20 | 0.20 | 1.05 | 0.71-1.54 | 0.82 | 0.97 |
| rs17466486 | 40328747 | A | 0.119 | 0.12 | 0.13 | 1.01 | 0.65-1.59 | 0.96 | 0.98 |
| rs11176052 | 40330098 | T | 0.314 | 0.32 | 0.27 | 0.71 | 0.49-1.02 | 0.066 | 0.28 |
| rs11564203 | 40330779 | A | 0.155 | 0.16 | 0.11 | 0.65 | 0.40-1.05 | 0.076 | 0.29 |
| rs7307562 | 40331158 | T | 0.380 | 0.39 | 0.32 | 0.76 | 0.55-1.05 | 0.096 | 0.34 |
| rs10506153 | 40331769 | C | 0.168 | 0.17 | 0.14 | 0.79 | 0.51-1.22 | 0.28 | 0.56 |
| rs963243 | 40331976 | C | 0.330 | 0.32 | 0.40 | 1.46 | 1.06-2.02 | 0.022 | 0.13 |
| rs11834950 | 40333796 | T | 0.167 | 0.17 | 0.14 | 0.80 | 0.52-1.24 | 0.32 | 0.61 |
| rs11829088 | 40333977 | G | 0.162 | 0.17 | 0.13 | 0.74 | 0.47-1.17 | 0.20 | 0.49 |
| rs2404834 | 40335205 | T | 0.136 | 0.14 | 0.14 | 0.97 | 0.62-1.49 | 0.87 | 0.97 |
| rs2404835 | 40336661 | T | 0.368 | 0.36 | 0.40 | 1.08 | 0.79-1.49 | 0.62 | 0.93 |
| rs4767971 | 40338230 | C | 0.144 | 0.13 | 0.23 | 2.00 | 1.37-2.94 | 0.00036 | **0.021** |
| rs1529376 | 40339034 | C | 0.013 | 0.01 | 0.02 | 1.04 | 0.28-3.86 | 0.96 | 0.98 |
| rs10735934 | 40339098 | C | 0.496 | 0.48 | 0.40 | 1.60 | 1.17-2.20 | 0.0033 | 0.079 |
| rs17491494 | 40339412 | A | 0.012 | 0.01 | 0.02 | 1.16 | 0.31-4.38 | 0.83 | 0.97 |
| rs17491536 | 40341862 | G | 0.064 | 0.07 | 0.04 | 0.54 | 0.25-1.18 | 0.12 | 0.39 |
| rs10506155 | 40342137 | A | 0.306 | 0.30 | 0.32 | 1.05 | 0.76-1.46 | 0.77 | 0.97 |
| rs919174 | 40342447 | T | 0.111 | 0.12 | 0.08 | 0.70 | 0.41-1.22 | 0.21 | 0.49 |
| rs4768231 | 40343381 | G | 0.136 | 0.12 | 0.23 | 2.03 | 1.38-2.99 | 0.00030 | **0.021** |
| rs10459264 | 40344206 | T | 0.096 | 0.10 | 0.08 | 0.65 | 0.36-1.19 | 0.17 | 0.46 |
| rs10878405 | 40348452 | A | 0.259 | 0.27 | 0.21 | 0.77 | 0.53-1.11 | 0.16 | 0.45 |
| rs11176143 | 40348561 | A | 0.077 | 0.08 | 0.05 | 0.60 | 0.30-1.22 | 0.16 | 0.45 |
| rs115625901 | 40349671 | T | 0.014 | 0.01 | 0.02 | 0.85 | 0.24-3.08 | 0.81 | 0.97 |
| rs7303525 | 40350973 | C | 0.179 | 0.18 | 0.19 | 1.07 | 0.72-1.60 | 0.73 | 0.97 |
| rs7132187 | 40351006 | A | 0.308 | 0.31 | 0.32 | 1.04 | 0.75-1.45 | 0.81 | 0.97 |
| rs7307310 | 40351379 | T | 0.142 | 0.13 | 0.23 | 1.94 | 1.32-2.85 | 0.00068 | **0.027** |
| rs11176153 | 40352036 | C | 0.179 | 0.18 | 0.19 | 1.07 | 0.72-1.60 | 0.73 | 0.97 |
| rs11176165 | 40354862 | C | 0.310 | 0.31 | 0.32 | 1.05 | 0.75-1.46 | 0.79 | 0.97 |
| rs11564146 | 40360506 | C | 0.060 | 0.06 | 0.08 | 1.39 | 0.76-2.55 | 0.29 | 0.56 |
| rs17466570 | 40360753 | T | 0.021 | 0.02 | 0.03 | 1.42 | 0.57-3.56 | 0.45 | 0.77 |
| rs11176195 | 40362405 | C | 0.095 | 0.10 | 0.07 | 0.61 | 0.33-1.12 | 0.11 | 0.38 |
| rs17444202 | 40362519 | T | 0.127 | 0.13 | 0.08 | 0.58 | 0.34-1.00 | 0.051 | 0.23 |
| rs33962975 | 40363528 | G | 0.122 | 0.12 | 0.12 | 0.95 | 0.59-1.52 | 0.82 | 0.97 |
| rs7134408 | 40363731 | A | 0.374 | 0.36 | 0.45 | 1.43 | 1.05-1.94 | 0.024 | 0.13 |
| rs11835105 | 40364243 | G | 0.210 | 0.22 | 0.15 | 0.60 | 0.39-0.93 | 0.022 | 0.13 |
| rs10878434 | 40364489 | A | 0.227 | 0.23 | 0.23 | 0.99 | 0.69-1.42 | 0.94 | 0.98 |
| rs3761863 | 40364850 | A | 0.367 | 0.36 | 0.46 | 1.51 | 1.11-2.05 | 0.0082 | 0.11 |
| rs10878441 | 40368129 | A | 0.371 | 0.36 | 0.46 | 1.48 | 1.09-2.01 | 0.013 | 0.11 |
| rs3886747 | 40368149 | C | 0.371 | 0.36 | 0.46 | 1.48 | 1.09-2.01 | 0.013 | 0.11 |
| rs10784548 | 40368744 | T | 0.371 | 0.36 | 0.46 | 1.48 | 1.09-2.01 | 0.013 | 0.11 |
| rs1465527 | 40369805 | C | 0.185 | 0.17 | 0.27 | 1.82 | 1.26-2.62 | 0.0013 | **0.039** |
| rs56820450 | 40371409 | G | 0.012 | 0.01 | 0.01 | 0.80 | 0.17-3.82 | 0.78 | 0.97 |
| rs10878452 | 40372798 | T | 0.108 | 0.11 | 0.09 | 0.64 | 0.36-1.13 | 0.12 | 0.39 |
| A1: reference allele; MAF: minor allele frequency; Fcontrol: low/moderate risk of ethanol dependence; Fcase: high risk of ethanol dependence; OR: odds ratio; 95% CI: 95% confidence interval; P: p-value (additive model); P_FDR_: False Discovery Rate (FDR)-adjusted p-value (Benjamini-Hochberg) | | | | | | | | | |
| Multivariate logistic regression - covariates: sex, age and principal component 1 (PC1) | | | | | | | | | |
| ^a^Human genome assembly: GRCh38 | | | | | | | | | |

| **Table S2. *LRRK2* locus: imputed variants evaluated in the discovery cohort (Bambuí, Brazil)** | | | | | | | | |
| --- | --- | --- | --- | --- | --- | --- | --- | --- |
| **SNP** | **Coordinate (chr12)^a^** | **A1** | **MAF** | **F_control_** | **F_case_** | **OR** | **95% CI** | **P** |
| rs17443178 | 40192263 | T | 0.01 | 0.012 | 0.005 | 0.40 | 0.05-3.18 | 0.39 |
| rs17443185 | 40192305 | A | 0.01 | 0.012 | 0.005 | 0.40 | 0.05-3.18 | 0.39 |
| rs17519258 | 40192381 | C | 0.03 | 0.027 | 0.030 | 1.21 | 0.48-3.06 | 0.68 |
| rs11175540 | 40192493 | A | 0.05 | 0.049 | 0.065 | 1.48 | 0.77-2.84 | 0.24 |
| rs17443199 | 40192631 | C | 0.15 | 0.145 | 0.160 | 1.17 | 0.75-1.81 | 0.49 |
| rs2131081 | 40192829 | C | 0.02 | 0.014 | 0.020 | 1.55 | 0.47-5.12 | 0.47 |
| rs17443206 | 40193212 | T | 0.01 | 0.012 | 0.005 | 0.40 | 0.05-3.18 | 0.39 |
| rs2254475 | 40193253 | C | 0.04 | 0.034 | 0.040 | 1.20 | 0.53-2.72 | 0.66 |
| rs113126072 | 40193671 | delG | 0.08 | 0.080 | 0.090 | 1.11 | 0.65-1.89 | 0.71 |
| rs17484003 | 40193719 | C | 0.03 | 0.026 | 0.025 | 0.97 | 0.35-2.69 | 0.95 |
| rs17465540 | 40193898 | G | 0.10 | 0.095 | 0.095 | 0.99 | 0.58-1.69 | 0.96 |
| rs1844921 | 40193900 | C | 0.41 | 0.412 | 0.385 | 0.92 | 0.67-1.27 | 0.61 |
| rs11175546 | 40194013 | C | 0.02 | 0.024 | 0.020 | 0.80 | 0.27-2.40 | 0.69 |
| rs202201521 | 40194087 | delCG | 0.01 | 0.011 | 0.015 | 2.09 | 0.55-7.93 | 0.28 |
| rs1491924 | 40194128 | C | 0.05 | 0.049 | 0.065 | 1.48 | 0.77-2.84 | 0.24 |
| rs7310104 | 40194788 | A | 0.29 | 0.291 | 0.320 | 1.16 | 0.82-1.62 | 0.41 |
| rs7975693 | 40194876 | T | 0.16 | 0.158 | 0.140 | 0.86 | 0.55-1.34 | 0.50 |
| rs10506146 | 40194893 | G | 0.02 | 0.014 | 0.020 | 1.55 | 0.47-5.12 | 0.47 |
| rs2046931 | 40195305 | A | 0.29 | 0.292 | 0.320 | 1.15 | 0.82-1.62 | 0.41 |
| rs76180339 | 40195404 | T | 0.08 | 0.080 | 0.090 | 1.11 | 0.65-1.89 | 0.71 |
| rs34041555 | 40196542 | A | 0.13 | 0.131 | 0.160 | 1.31 | 0.84-2.04 | 0.23 |
| rs11564274 | 40196818 | T | 0.09 | 0.092 | 0.105 | 1.08 | 0.65-1.79 | 0.76 |
| rs1491923 | 40197315 | G | 0.29 | 0.291 | 0.320 | 1.15 | 0.82-1.62 | 0.43 |
| rs1491922 | 40197371 | C | 0.29 | 0.292 | 0.320 | 1.14 | 0.81-1.60 | 0.45 |
| rs11564212 | 40197810 | T | 0.03 | 0.028 | 0.025 | 0.93 | 0.34-2.54 | 0.89 |
| rs79410089 | 40198262 | G | 0.08 | 0.082 | 0.080 | 0.96 | 0.55-1.68 | 0.89 |
| rs4767964 | 40198466 | T | 0.02 | 0.014 | 0.020 | 1.55 | 0.47-5.12 | 0.47 |
| rs4767965 | 40198538 | A | 0.03 | 0.028 | 0.025 | 0.93 | 0.34-2.54 | 0.89 |
| rs2708419 | 40198586 | C | 0.42 | 0.412 | 0.440 | 1.06 | 0.78-1.44 | 0.72 |
| rs76228370 | 40198609 | T | 0.02 | 0.014 | 0.030 | 1.85 | 0.68-5.05 | 0.23 |
| rs201212381 | 40198693 | dupT | 0.03 | 0.028 | 0.025 | 0.93 | 0.34-2.54 | 0.89 |
| rs2708420 | 40198699 | G | 0.40 | 0.396 | 0.409 | 1.02 | 0.74-1.39 | 0.92 |
| rs2708422 | 40199874 | T | 0.40 | 0.399 | 0.410 | 1.00 | 0.73-1.37 | 1.00 |
| rs17465610 | 40200117 | T | 0.03 | 0.028 | 0.025 | 0.93 | 0.34-2.54 | 0.89 |
| rs180695044 | 40200337 | G | 0.02 | 0.025 | 0.018 | 0.99 | 0.32-3.08 | 0.99 |
| rs2708423 | 40200371 | G | 0.40 | 0.399 | 0.410 | 1.00 | 0.73-1.37 | 1.00 |
| rs2708424 | 40200768 | C | 0.38 | 0.383 | 0.375 | 0.94 | 0.68-1.29 | 0.70 |
| rs2638233 | 40200920 | C | 0.40 | 0.399 | 0.410 | 1.00 | 0.73-1.37 | 1.00 |
| rs140949659 | 40201172 | delA | 0.05 | 0.050 | 0.035 | 0.69 | 0.30-1.59 | 0.38 |
| rs2723266 | 40201740 | C | 0.01 | 0.011 | 0.015 | 1.35 | 0.37-4.89 | 0.65 |
| rs147973391 | 40201919 | A | 0.02 | 0.025 | 0.018 | 0.99 | 0.32-3.08 | 0.99 |
| rs1491934 | 40202364 | A | 0.11 | 0.110 | 0.095 | 0.87 | 0.51-1.47 | 0.60 |
| rs2638274 | 40203345 | G | 0.09 | 0.083 | 0.138 | 1.92 | 1.17-3.12 | 0.0092 |
| rs138053637 | 40203477 | C | 0.01 | 0.014 | 0.005 | 0.38 | 0.05-2.95 | 0.35 |
| rs115515759 | 40203707 | A | 0.01 | 0.011 | 0.015 | 1.16 | 0.31-4.38 | 0.83 |
| rs4768221 | 40203743 | G | 0.38 | 0.387 | 0.340 | 0.85 | 0.61-1.17 | 0.31 |
| rs117762348 | 40203810 | G | 0.07 | 0.073 | 0.055 | 0.69 | 0.35-1.34 | 0.28 |
| rs4767967 | 40204067 | C | 0.38 | 0.386 | 0.337 | 0.84 | 0.60-1.16 | 0.28 |
| rs11564273 | 40204350 | G | 0.07 | 0.073 | 0.055 | 0.69 | 0.35-1.34 | 0.28 |
| rs1491940 | 40204542 | A | 0.43 | 0.435 | 0.427 | 0.99 | 0.73-1.35 | 0.97 |
| rs2708426 | 40205069 | T | 0.09 | 0.083 | 0.131 | 1.82 | 1.12-2.98 | 0.017 |
| rs2708427 | 40205137 | C | 0.43 | 0.435 | 0.427 | 0.99 | 0.73-1.35 | 0.97 |
| rs10506147 | 40205596 | C | 0.03 | 0.028 | 0.025 | 0.93 | 0.34-2.54 | 0.89 |
| rs5797662 | 40205813 | dupT | 0.34 | 0.339 | 0.329 | 0.94 | 0.66-1.33 | 0.73 |
| rs2723259 | 40206894 | G | 0.40 | 0.399 | 0.410 | 1.00 | 0.73-1.37 | 1.00 |
| rs2131086 | 40207140 | C | 0.50 | 0.500 | 0.490 | 0.91 | 0.67-1.24 | 0.54 |
| rs11564190 | 40207298 | G | 0.02 | 0.014 | 0.020 | 1.55 | 0.47-5.12 | 0.47 |
| rs12305120 | 40209519 | C | 0.02 | 0.016 | 0.030 | 1.62 | 0.62-4.20 | 0.33 |
| rs76524269 | 40209547 | C | 0.02 | 0.014 | 0.020 | 1.55 | 0.47-5.12 | 0.47 |
| rs118075840 | 40209874 | T | 0.03 | 0.026 | 0.025 | 0.93 | 0.34-2.53 | 0.88 |
| rs2708429 | 40210155 | T | 0.01 | 0.011 | 0.015 | 1.48 | 0.41-5.39 | 0.55 |
| rs2638229 | 40210266 | A | 0.43 | 0.435 | 0.428 | 0.99 | 0.73-1.34 | 0.95 |
| rs2638230 | 40210273 | T | 0.40 | 0.405 | 0.399 | 0.98 | 0.72-1.34 | 0.90 |
| rs10784428 | 40210806 | A | 0.40 | 0.408 | 0.355 | 0.81 | 0.59-1.12 | 0.20 |
| rs2172240 | 40211019 | A | 0.09 | 0.084 | 0.140 | 1.98 | 1.23-3.19 | 0.0052 |
| rs201152187 | 40211088 | delA | 0.03 | 0.029 | 0.025 | 0.87 | 0.32-2.37 | 0.78 |
| rs76664306 | 40211137 | A | 0.01 | 0.014 | 0.005 | 0.38 | 0.05-2.94 | 0.35 |
| rs2131079 | 40211325 | A | 0.03 | 0.028 | 0.025 | 0.93 | 0.34-2.54 | 0.89 |
| rs2131080 | 40211326 | T | 0.03 | 0.028 | 0.025 | 0.93 | 0.34-2.54 | 0.89 |
| rs17443304 | 40211402 | G | 0.01 | 0.013 | 0.005 | 0.43 | 0.05-3.46 | 0.43 |
| rs76826991 | 40211724 | T | 0.07 | 0.072 | 0.055 | 0.70 | 0.36-1.36 | 0.30 |
| rs17490536 | 40212101 | T | 0.01 | 0.011 | 0.015 | 1.17 | 0.31-4.42 | 0.82 |
| rs2263419 | 40212114 | G | 0.40 | 0.400 | 0.410 | 1.00 | 0.73-1.36 | 0.98 |
| rs2172241 | 40212199 | T | 0.09 | 0.084 | 0.140 | 1.98 | 1.23-3.19 | 0.0052 |
| rs2263420 | 40212205 | C | 0.40 | 0.400 | 0.410 | 1.00 | 0.73-1.36 | 0.98 |
| rs59934346 | 40212354 | C | 0.02 | 0.014 | 0.025 | 1.46 | 0.49-4.29 | 0.49 |
| rs138259753 | 40212546 | G | 0.02 | 0.014 | 0.020 | 1.55 | 0.47-5.12 | 0.47 |
| rs1907630 | 40212750 | A | 0.09 | 0.084 | 0.140 | 1.98 | 1.23-3.19 | 0.0052 |
| rs1907631 | 40212861 | C | 0.09 | 0.084 | 0.140 | 1.98 | 1.23-3.19 | 0.0052 |
| rs17519419 | 40212900 | T | 0.11 | 0.108 | 0.105 | 0.90 | 0.53-1.53 | 0.69 |
| rs17519426 | 40213080 | G | 0.02 | 0.021 | 0.025 | 1.00 | 0.36-2.81 | 0.99 |
| rs75028169 | 40213097 | A | 0.02 | 0.014 | 0.020 | 1.55 | 0.47-5.12 | 0.47 |
| rs17465681 | 40213190 | A | 0.07 | 0.072 | 0.055 | 0.70 | 0.36-1.36 | 0.30 |
| rs2201141 | 40213296 | G | 0.40 | 0.400 | 0.410 | 1.00 | 0.73-1.36 | 0.98 |
| rs2201142 | 40213423 | A | 0.27 | 0.266 | 0.265 | 0.96 | 0.68-1.37 | 0.84 |
| rs73102291 | 40213708 | A | 0.10 | 0.102 | 0.081 | 0.80 | 0.44-1.43 | 0.45 |
| rs1388594 | 40213845 | C | 0.49 | 0.488 | 0.495 | 1.09 | 0.80-1.48 | 0.57 |
| rs76906269 | 40213907 | G | 0.01 | 0.014 | 0.005 | 0.38 | 0.05-2.95 | 0.35 |
| rs7973254 | 40213964 | C | 0.37 | 0.372 | 0.360 | 0.92 | 0.67-1.26 | 0.59 |
| rs143510558 | 40214040 | delG | 0.02 | 0.014 | 0.020 | 1.55 | 0.47-5.12 | 0.47 |
| rs7970326 | 40214089 | C | 0.37 | 0.372 | 0.360 | 0.92 | 0.67-1.27 | 0.61 |
| rs115712145 | 40214516 | G | 0.01 | 0.011 | 0.015 | 1.17 | 0.31-4.42 | 0.82 |
| rs17461575 | 40215778 | T | 0.03 | 0.028 | 0.025 | 0.93 | 0.34-2.53 | 0.88 |
| rs11564107 | 40215948 | G | 0.03 | 0.028 | 0.025 | 0.93 | 0.34-2.53 | 0.88 |
| rs17443345 | 40216083 | T | 0.09 | 0.093 | 0.095 | 1.02 | 0.59-1.74 | 0.95 |
| rs7964154 | 40216366 | T | 0.02 | 0.016 | 0.030 | 1.49 | 0.57-3.86 | 0.42 |
| rs28365206 | 40216587 | A | 0.02 | 0.014 | 0.020 | 1.55 | 0.47-5.12 | 0.47 |
| rs149124411 | 40216942 | T | 0.02 | 0.018 | 0.010 | 0.56 | 0.14-2.34 | 0.43 |
| rs2201144 | 40217061 | C | 0.07 | 0.065 | 0.135 | 2.04 | 1.32-3.15 | 0.0014 |
| rs11175620 | 40217062 | C | 0.10 | 0.099 | 0.075 | 0.69 | 0.39-1.22 | 0.20 |
| rs149397425 | 40217727 | delCC | 0.10 | 0.099 | 0.075 | 0.70 | 0.39-1.23 | 0.21 |
| rs4291738 | 40217816 | T | 0.10 | 0.102 | 0.100 | 0.92 | 0.54-1.59 | 0.78 |
| rs117408408 | 40217868 | G | 0.01 | 0.014 | 0.005 | 0.38 | 0.05-2.94 | 0.35 |
| rs11386076 | 40218361 | delT | 0.05 | 0.049 | 0.056 | 1.24 | 0.60-2.57 | 0.56 |
| rs11564131 | 40218408 | G | 0.02 | 0.014 | 0.020 | 1.55 | 0.47-5.12 | 0.47 |
| rs75695609 | 40219405 | A | 0.01 | 0.011 | 0.015 | 1.17 | 0.31-4.42 | 0.82 |
| rs7975769 | 40219749 | C | 0.02 | 0.016 | 0.030 | 1.49 | 0.57-3.86 | 0.42 |
| rs1602578 | 40220195 | G | 0.39 | 0.394 | 0.345 | 0.85 | 0.62-1.17 | 0.32 |
| rs117528165 | 40220260 | T | 0.01 | 0.014 | 0.005 | 0.38 | 0.05-2.94 | 0.35 |
| rs1907634 | 40220518 | A | 0.07 | 0.058 | 0.120 | 2.47 | 1.48-4.12 | 0.00056 |
| rs199541433 | 40220762 | dupT | 0.03 | 0.028 | 0.026 | 0.95 | 0.35-2.61 | 0.93 |
| rs10878224 | 40220799 | T | 0.38 | 0.387 | 0.330 | 0.81 | 0.59-1.12 | 0.20 |
| rs2708435 | 40220854 | A | 0.42 | 0.420 | 0.430 | 0.98 | 0.72-1.34 | 0.92 |
| rs2708436 | 40220944 | A | 0.07 | 0.058 | 0.120 | 2.47 | 1.48-4.12 | 0.00056 |
| rs2708437 | 40220977 | T | 0.07 | 0.058 | 0.120 | 2.47 | 1.48-4.12 | 0.00056 |
| rs142294452 | 40221123 | dupA | 0.11 | 0.109 | 0.089 | 0.71 | 0.41-1.23 | 0.22 |
| rs10748014 | 40221267 | T | 0.38 | 0.385 | 0.330 | 0.81 | 0.59-1.12 | 0.20 |
| rs2708438 | 40221630 | G | 0.07 | 0.058 | 0.120 | 2.47 | 1.48-4.12 | 0.00056 |
| rs144157802 | 40222269 | dupCA | 0.03 | 0.029 | 0.030 | 1.08 | 0.43-2.72 | 0.88 |
| rs1388598 | 40222364 | A | 0.11 | 0.107 | 0.105 | 0.91 | 0.53-1.55 | 0.72 |
| rs1472117 | 40222415 | T | 0.07 | 0.058 | 0.120 | 2.47 | 1.48-4.12 | 0.00056 |
| rs7294619 | 40223400 | C | 0.10 | 0.099 | 0.075 | 0.70 | 0.39-1.23 | 0.21 |
| rs201496042 | 40223721 | delAAAGC | 0.02 | 0.014 | 0.020 | 1.55 | 0.47-5.12 | 0.47 |
| rs10878226 | 40223890 | C | 0.10 | 0.099 | 0.075 | 0.70 | 0.39-1.23 | 0.21 |
| rs141560291 | 40223957 | C | 0.01 | 0.012 | 0.015 | 1.64 | 0.44-6.18 | 0.46 |
| rs1491945 | 40224610 | A | 0.02 | 0.014 | 0.020 | 1.55 | 0.47-5.12 | 0.47 |
| rs186923145 | 40224858 | T | 0.02 | 0.014 | 0.020 | 1.55 | 0.47-5.12 | 0.47 |
| rs58621298 | 40224884 | C | 0.02 | 0.021 | 0.025 | 1.00 | 0.36-2.81 | 0.99 |
| rs139549144 | 40224912 | G | 0.03 | 0.028 | 0.025 | 0.93 | 0.34-2.55 | 0.89 |
| rs112643657 | 40225008 | C | 0.02 | 0.021 | 0.025 | 1.00 | 0.36-2.81 | 0.99 |
| rs2723273 | 40225499 | G | 0.01 | 0.011 | 0.015 | 1.48 | 0.41-5.39 | 0.55 |
| rs17465737 | 40225955 | C | 0.02 | 0.016 | 0.020 | 1.01 | 0.28-3.58 | 0.99 |
| rs1491943 | 40226614 | T | 0.10 | 0.105 | 0.101 | 0.90 | 0.53-1.55 | 0.72 |
| rs144635285 | 40226953 | delC | 0.03 | 0.029 | 0.030 | 0.94 | 0.39-2.29 | 0.90 |
| rs1491942 | 40227006 | G | 0.21 | 0.207 | 0.253 | 1.28 | 0.90-1.82 | 0.17 |
| rs1491941 | 40227013 | G | 0.40 | 0.395 | 0.402 | 1.00 | 0.73-1.37 | 0.98 |
| rs138873140 | 40227176 | G | 0.02 | 0.014 | 0.020 | 1.55 | 0.47-5.12 | 0.47 |
| rs149406366 | 40227354 | A | 0.03 | 0.028 | 0.025 | 0.93 | 0.34-2.55 | 0.89 |
| rs11175645 | 40228090 | G | 0.10 | 0.099 | 0.075 | 0.70 | 0.39-1.23 | 0.21 |
| rs4567538 | 40228102 | T | 0.37 | 0.368 | 0.356 | 0.94 | 0.68-1.29 | 0.69 |
| rs150540643 | 40228666 | A | 0.03 | 0.028 | 0.025 | 0.93 | 0.34-2.55 | 0.89 |
| rs149720743 | 40228763 | C | 0.01 | 0.013 | 0.020 | 1.87 | 0.56-6.22 | 0.31 |
| rs75143074 | 40229522 | C | 0.01 | 0.014 | 0.005 | 0.38 | 0.05-2.97 | 0.36 |
| rs11175656 | 40229953 | A | 0.38 | 0.390 | 0.332 | 0.80 | 0.58-1.11 | 0.18 |
| rs11175658 | 40230508 | G | 0.10 | 0.101 | 0.075 | 0.68 | 0.38-1.21 | 0.19 |
| rs74324737 | 40231279 | A | 0.10 | 0.101 | 0.075 | 0.68 | 0.38-1.21 | 0.19 |
| rs2708441 | 40231365 | A | 0.06 | 0.058 | 0.121 | 2.50 | 1.50-4.17 | 0.00046 |
| rs10631840 | 40231391 | delAAA | 0.20 | 0.190 | 0.253 | 1.53 | 1.03-2.27 | 0.037 |
| rs199832313 | 40231794 | dupTA | 0.03 | 0.028 | 0.025 | 0.93 | 0.34-2.54 | 0.89 |
| rs116644875 | 40231807 | G | 0.10 | 0.102 | 0.075 | 0.66 | 0.37-1.18 | 0.16 |
| rs148217035 | 40231996 | T | 0.01 | 0.010 | 0.035 | 5.51 | 1.92-15.76 | 0.0015 |
| rs1352878 | 40232483 | C | 0.03 | 0.026 | 0.045 | 1.57 | 0.73-3.40 | 0.25 |
| rs1352877 | 40232576 | T | 0.03 | 0.028 | 0.051 | 2.16 | 0.98-4.76 | 0.056 |
| rs139999912 | 40233122 | G | 0.01 | 0.011 | 0.015 | 1.17 | 0.31-4.42 | 0.82 |
| rs1817817 | 40233202 | A | 0.06 | 0.057 | 0.121 | 2.51 | 1.50-4.19 | 0.00041 |
| rs117544909 | 40233654 | T | 0.01 | 0.014 | 0.005 | 0.38 | 0.05-2.97 | 0.36 |
| rs150082974 | 40234430 | A | 0.04 | 0.037 | 0.025 | 0.73 | 0.28-1.92 | 0.52 |
| rs11175666 | 40234631 | T | 0.10 | 0.100 | 0.075 | 0.69 | 0.39-1.23 | 0.21 |
| rs2723268 | 40234784 | G | 0.06 | 0.057 | 0.121 | 2.51 | 1.50-4.19 | 0.00041 |
| rs75759934 | 40235553 | delA | 0.02 | 0.014 | 0.020 | 1.55 | 0.47-5.12 | 0.47 |
| rs2131088 | 40235752 | T | 0.06 | 0.057 | 0.121 | 2.51 | 1.50-4.19 | 0.00041 |
| rs377568728 | 40235795 | G | 0.18 | 0.190 | 0.128 | 0.60 | 0.35-1.02 | 0.061 |
| rs117297508 | 40235945 | T | 0.03 | 0.028 | 0.025 | 0.93 | 0.34-2.55 | 0.89 |
| rs2723269 | 40235985 | T | 0.06 | 0.057 | 0.121 | 2.51 | 1.50-4.19 | 0.00041 |
| rs11564115 | 40236837 | G | 0.03 | 0.028 | 0.070 | 3.25 | 1.63-6.50 | 0.00083 |
| rs2249281 | 40237556 | T | 0.03 | 0.029 | 0.036 | 1.08 | 0.47-2.49 | 0.86 |
| rs10878244 | 40237806 | A | 0.14 | 0.138 | 0.138 | 0.96 | 0.62-1.49 | 0.87 |
| rs10878246 | 40238297 | G | 0.16 | 0.158 | 0.150 | 0.93 | 0.61-1.40 | 0.72 |
| rs10506148 | 40238792 | A | 0.03 | 0.028 | 0.070 | 3.31 | 1.65-6.62 | 0.00072 |
| rs17519531 | 40238959 | T | 0.01 | 0.009 | 0.025 | 3.29 | 1.02-10.58 | 0.046 |
| rs954884 | 40239156 | T | 0.48 | 0.430 | 0.473 | 1.46 | 1.08-1.97 | 0.015 |
| rs10784444 | 40239686 | A | 0.48 | 0.430 | 0.473 | 1.46 | 1.08-1.97 | 0.015 |
| rs201655125 | 40239796 | delA | 0.03 | 0.031 | 0.025 | 0.83 | 0.31-2.26 | 0.72 |
| rs11564187 | 40240401 | G | 0.03 | 0.031 | 0.025 | 0.83 | 0.31-2.26 | 0.72 |
| rs17484141 | 40240672 | A | 0.02 | 0.014 | 0.020 | 1.55 | 0.47-5.12 | 0.47 |
| rs11564210 | 40241017 | T | 0.03 | 0.031 | 0.025 | 0.83 | 0.31-2.26 | 0.72 |
| rs13328957 | 40241113 | G | 0.01 | 0.011 | 0.010 | 0.93 | 0.19-4.46 | 0.92 |
| rs17519573 | 40241554 | A | 0.16 | 0.159 | 0.150 | 0.93 | 0.61-1.40 | 0.71 |
| rs17519580 | 40243361 | C | 0.02 | 0.016 | 0.030 | 2.05 | 0.77-5.41 | 0.15 |
| rs144696935 | 40244894 | C | 0.02 | 0.014 | 0.020 | 1.55 | 0.47-5.12 | 0.47 |
| rs150364407 | 40245873 | T | 0.02 | 0.014 | 0.020 | 1.55 | 0.47-5.12 | 0.47 |
| rs2404581 | 40246099 | T | 0.50 | 0.489 | 0.410 | 0.68 | 0.50-0.93 | 0.015 |
| rs187455350 | 40246160 | G | 0.02 | 0.016 | 0.005 | 0.31 | 0.04-2.37 | 0.26 |
| rs1388599 | 40246358 | C | 0.09 | 0.086 | 0.090 | 1.04 | 0.62-1.76 | 0.87 |
| rs148547517 | 40246537 | G | 0.02 | 0.016 | 0.005 | 0.31 | 0.04-2.37 | 0.26 |
| rs73106349 | 40246556 | G | 0.02 | 0.016 | 0.030 | 2.05 | 0.77-5.41 | 0.15 |
| rs1388596 | 40246824 | G | 0.08 | 0.071 | 0.140 | 2.42 | 1.49-3.94 | 0.00071 |
| rs10544982 | 40247430 | dupACT | 0.44 | 0.429 | 0.475 | 1.48 | 1.09-2.01 | 0.013 |
| rs10450793 | 40247505 | A | 0.02 | 0.017 | 0.030 | 2.14 | 0.81-5.62 | 0.12 |
| rs201595811 | 40247524 | insTTTATACA | 0.38 | 0.371 | 0.455 | 1.41 | 1.03-1.94 | 0.031 |
| rs732374 | 40249666 | T | 0.31 | 0.323 | 0.230 | 0.67 | 0.48-0.95 | 0.023 |
| rs10878262 | 40250477 | C | 0.31 | 0.323 | 0.230 | 0.67 | 0.48-0.95 | 0.023 |
| rs12371502 | 40250503 | C | 0.47 | 0.481 | 0.385 | 0.70 | 0.51-0.95 | 0.021 |
| rs11175729 | 40250565 | T | 0.17 | 0.173 | 0.180 | 1.06 | 0.71-1.59 | 0.77 |
| rs17443407 | 40250701 | T | 0.09 | 0.089 | 0.085 | 0.91 | 0.51-1.61 | 0.75 |
| rs117502843 | 40250753 | A | 0.02 | 0.016 | 0.005 | 0.31 | 0.04-2.37 | 0.26 |
| rs17443414 | 40250950 | G | 0.09 | 0.091 | 0.090 | 0.95 | 0.54-1.67 | 0.86 |
| rs7134379 | 40251072 | T | 0.27 | 0.265 | 0.270 | 1.02 | 0.73-1.44 | 0.89 |
| rs1491939 | 40251794 | G | 0.11 | 0.105 | 0.185 | 2.03 | 1.31-3.14 | 0.0014 |
| rs117203131 | 40251935 | A | 0.02 | 0.016 | 0.005 | 0.31 | 0.04-2.37 | 0.26 |
| rs12422796 | 40252732 | A | 0.03 | 0.031 | 0.025 | 0.83 | 0.31-2.26 | 0.72 |
| rs7969677 | 40253146 | A | 0.17 | 0.171 | 0.175 | 1.04 | 0.69-1.56 | 0.85 |
| rs1463717 | 40253843 | T | 0.03 | 0.031 | 0.025 | 0.83 | 0.31-2.26 | 0.72 |
| rs150645252 | 40254154 | T | 0.02 | 0.016 | 0.005 | 0.31 | 0.04-2.37 | 0.26 |
| rs142152465 | 40254448 | A | 0.02 | 0.017 | 0.010 | 0.63 | 0.15-2.60 | 0.52 |
| rs146375916 | 40254481 | T | 0.01 | 0.010 | 0.010 | 1.23 | 0.25-5.96 | 0.80 |
| rs2723265 | 40255521 | C | 0.03 | 0.031 | 0.029 | 0.80 | 0.33-1.97 | 0.63 |
| rs7132171 | 40255947 | C | 0.46 | 0.472 | 0.396 | 0.74 | 0.54-1.01 | 0.055 |
| rs10784451 | 40256086 | A | 0.17 | 0.173 | 0.187 | 1.09 | 0.73-1.63 | 0.67 |
| rs10784452 | 40256173 | A | 0.30 | 0.297 | 0.316 | 1.04 | 0.74-1.46 | 0.81 |
| rs147831725 | 40256303 | A | 0.03 | 0.031 | 0.025 | 0.83 | 0.31-2.26 | 0.72 |
| rs1491936 | 40256647 | C | 0.14 | 0.139 | 0.115 | 0.80 | 0.49-1.28 | 0.35 |
| rs1907632 | 40256700 | A | 0.14 | 0.143 | 0.125 | 0.85 | 0.54-1.33 | 0.48 |
| rs11564185 | 40256915 | T | 0.03 | 0.031 | 0.025 | 0.83 | 0.31-2.26 | 0.72 |
| rs200586155 | 40256998 | delACT | 0.01 | 0.012 | 0.015 | 1.47 | 0.39-5.46 | 0.57 |
| rs11564209 | 40257073 | T | 0.03 | 0.031 | 0.025 | 0.83 | 0.31-2.26 | 0.72 |
| rs17461664 | 40258170 | T | 0.03 | 0.031 | 0.025 | 0.83 | 0.31-2.26 | 0.72 |
| rs2046928 | 40258592 | G | 0.12 | 0.115 | 0.126 | 1.00 | 0.64-1.57 | 0.99 |
| rs17490796 | 40260073 | T | 0.01 | 0.010 | 0.010 | 0.79 | 0.16-3.93 | 0.78 |
| rs17465877 | 40260169 | A | 0.01 | 0.012 | 0.015 | 1.47 | 0.39-5.46 | 0.57 |
| rs11564152 | 40261071 | G | 0.03 | 0.031 | 0.025 | 0.83 | 0.30-2.25 | 0.71 |
| rs11175766 | 40261214 | C | 0.12 | 0.115 | 0.130 | 1.03 | 0.66-1.61 | 0.89 |
| rs17465898 | 40262362 | G | 0.03 | 0.027 | 0.015 | 0.40 | 0.12-1.37 | 0.14 |
| rs17465912 | 40262593 | T | 0.14 | 0.140 | 0.105 | 0.71 | 0.43-1.16 | 0.17 |
| rs17443552 | 40262875 | A | 0.14 | 0.143 | 0.115 | 0.77 | 0.48-1.25 | 0.30 |
| rs17490817 | 40263160 | A | 0.12 | 0.118 | 0.110 | 0.88 | 0.53-1.45 | 0.61 |
| rs11430638 | 40263402 | insA | 0.13 | 0.125 | 0.185 | 1.61 | 1.07-2.42 | 0.022 |
| rs10784461 | 40263735 | G | 0.40 | 0.410 | 0.325 | 0.74 | 0.54-1.03 | 0.072 |
| rs7308720 | 40263898 | G | 0.12 | 0.118 | 0.110 | 0.88 | 0.53-1.45 | 0.61 |
| rs146794882 | 40264251 | C | 0.03 | 0.030 | 0.025 | 0.75 | 0.28-2.01 | 0.56 |
| rs17465946 | 40264353 | A | 0.02 | 0.016 | 0.005 | 0.31 | 0.04-2.37 | 0.26 |
| rs10878284 | 40264403 | A | 0.18 | 0.178 | 0.192 | 1.06 | 0.71-1.56 | 0.79 |
| rs11175775 | 40264453 | C | 0.26 | 0.265 | 0.210 | 0.81 | 0.56-1.17 | 0.26 |
| rs17461692 | 40264614 | A | 0.02 | 0.014 | 0.020 | 1.55 | 0.47-5.12 | 0.47 |
| rs17443600 | 40265065 | T | 0.03 | 0.031 | 0.025 | 0.83 | 0.31-2.26 | 0.72 |
| rs17443607 | 40265162 | T | 0.03 | 0.035 | 0.025 | 0.54 | 0.20-1.45 | 0.22 |
| rs11175784 | 40265732 | T | 0.39 | 0.396 | 0.315 | 0.74 | 0.54-1.03 | 0.078 |
| rs7313276 | 40265847 | T | 0.09 | 0.089 | 0.077 | 0.87 | 0.49-1.54 | 0.63 |
| rs186675266 | 40265856 | A | 0.03 | 0.026 | 0.030 | 1.28 | 0.51-3.23 | 0.60 |
| rs17490865 | 40265904 | G | 0.10 | 0.100 | 0.076 | 0.64 | 0.35-1.17 | 0.15 |
| rs17490872 | 40266105 | A | 0.01 | 0.010 | 0.010 | 0.80 | 0.16-3.94 | 0.78 |
| rs10878290 | 40266273 | T | 0.31 | 0.304 | 0.400 | 1.55 | 1.12-2.13 | 0.0074 |
| rs11175787 | 40266292 | A | 0.40 | 0.406 | 0.323 | 0.74 | 0.54-1.03 | 0.073 |
| rs10878291 | 40266594 | C | 0.14 | 0.131 | 0.197 | 1.67 | 1.12-2.48 | 0.012 |
| rs17461720 | 40266685 | G | 0.03 | 0.035 | 0.025 | 0.54 | 0.20-1.45 | 0.22 |
| rs12317068 | 40266815 | A | 0.32 | 0.307 | 0.400 | 1.53 | 1.12-2.11 | 0.0085 |
| rs111341533 | 40266936 | G | 0.14 | 0.141 | 0.136 | 0.99 | 0.64-1.52 | 0.95 |
| rs144463374 | 40267078 | T | 0.04 | 0.044 | 0.030 | 0.77 | 0.33-1.85 | 0.56 |
| rs73108329 | 40267251 | T | 0.10 | 0.100 | 0.076 | 0.64 | 0.35-1.17 | 0.15 |
| rs12426891 | 40267479 | C | 0.12 | 0.119 | 0.105 | 0.76 | 0.46-1.25 | 0.28 |
| rs79833858 | 40267547 | A | 0.01 | 0.012 | 0.020 | 1.47 | 0.46-4.76 | 0.52 |
| rs4768224 | 40267601 | A | 0.31 | 0.304 | 0.400 | 1.55 | 1.12-2.13 | 0.0075 |
| rs144202154 | 40268889 | G | 0.01 | 0.016 | 0.005 | 0.34 | 0.04-2.66 | 0.31 |
| rs12146857 | 40270116 | G | 0.25 | 0.251 | 0.200 | 0.81 | 0.55-1.18 | 0.27 |
| rs11564207 | 40270841 | A | 0.14 | 0.145 | 0.116 | 0.77 | 0.48-1.24 | 0.29 |
| rs7308193 | 40271425 | G | 0.32 | 0.313 | 0.399 | 1.51 | 1.09-2.09 | 0.013 |
| rs11175814 | 40271652 | G | 0.01 | 0.015 | 0.005 | 0.41 | 0.05-3.27 | 0.40 |
| rs73108339 | 40272118 | T | 0.05 | 0.058 | 0.030 | 0.49 | 0.20-1.18 | 0.11 |
| rs17490921 | 40272580 | C | 0.02 | 0.018 | 0.025 | 1.12 | 0.40-3.11 | 0.83 |
| rs17443656 | 40272935 | A | 0.03 | 0.031 | 0.025 | 0.83 | 0.31-2.26 | 0.72 |
| rs10506150 | 40272958 | T | 0.07 | 0.078 | 0.040 | 0.51 | 0.24-1.08 | 0.077 |
| rs76660489 | 40273003 | delAC | 0.10 | 0.100 | 0.076 | 0.64 | 0.35-1.17 | 0.15 |
| rs10878299 | 40273073 | G | 0.12 | 0.118 | 0.105 | 0.76 | 0.46-1.26 | 0.28 |
| rs17466016 | 40273861 | A | 0.03 | 0.031 | 0.025 | 0.83 | 0.31-2.26 | 0.72 |
| rs10878302 | 40276024 | A | 0.12 | 0.119 | 0.105 | 0.76 | 0.46-1.27 | 0.30 |
| rs17443670 | 40276067 | A | 0.03 | 0.024 | 0.030 | 1.51 | 0.59-3.85 | 0.39 |
| rs4628750 | 40276254 | G | 0.03 | 0.031 | 0.025 | 0.83 | 0.31-2.26 | 0.72 |
| rs7971935 | 40276592 | A | 0.11 | 0.109 | 0.116 | 1.01 | 0.63-1.60 | 0.98 |
| rs141409298 | 40276792 | C | 0.03 | 0.031 | 0.025 | 0.83 | 0.31-2.26 | 0.72 |
| rs11175836 | 40276874 | T | 0.25 | 0.251 | 0.202 | 0.82 | 0.56-1.21 | 0.31 |
| rs4272849 | 40276999 | T | 0.38 | 0.393 | 0.310 | 0.73 | 0.53-1.02 | 0.067 |
| rs10784485 | 40277068 | C | 0.12 | 0.118 | 0.105 | 0.76 | 0.46-1.26 | 0.28 |
| rs7975730 | 40277480 | A | 0.01 | 0.010 | 0.010 | 0.80 | 0.16-3.94 | 0.78 |
| rs12320863 | 40277616 | A | 0.01 | 0.012 | 0.020 | 1.47 | 0.46-4.76 | 0.52 |
| rs12423551 | 40278792 | G | 0.02 | 0.015 | 0.020 | 1.52 | 0.46-5.01 | 0.50 |
| rs150574014 | 40279308 | delGTTAA | 0.03 | 0.031 | 0.025 | 0.83 | 0.31-2.26 | 0.72 |
| rs7309197 | 40279458 | A | 0.39 | 0.396 | 0.310 | 0.73 | 0.52-1.02 | 0.062 |
| rs7134572 | 40279566 | G | 0.14 | 0.132 | 0.197 | 1.66 | 1.12-2.48 | 0.012 |
| rs17519825 | 40279615 | T | 0.01 | 0.010 | 0.010 | 0.80 | 0.16-3.94 | 0.78 |
| rs75083520 | 40279711 | T | 0.03 | 0.029 | 0.020 | 0.61 | 0.20-1.82 | 0.37 |
| rs11564271 | 40279722 | G | 0.03 | 0.031 | 0.025 | 0.83 | 0.31-2.26 | 0.72 |
| rs11564181 | 40279737 | A | 0.01 | 0.015 | 0.010 | 0.74 | 0.16-3.39 | 0.70 |
| rs11175843 | 40280237 | C | 0.18 | 0.179 | 0.195 | 1.13 | 0.76-1.67 | 0.54 |
| rs7488873 | 40280792 | T | 0.32 | 0.313 | 0.400 | 1.52 | 1.10-2.10 | 0.012 |
| rs149331112 | 40280832 | T | 0.01 | 0.015 | 0.010 | 0.74 | 0.16-3.38 | 0.70 |
| rs56136805 | 40281023 | A | 0.02 | 0.018 | 0.025 | 1.12 | 0.40-3.11 | 0.83 |
| rs58072940 | 40281076 | insC | 0.18 | 0.179 | 0.152 | 0.87 | 0.54-1.41 | 0.57 |
| rs11175845 | 40281250 | G | 0.39 | 0.396 | 0.310 | 0.73 | 0.52-1.02 | 0.062 |
| rs17466044 | 40281544 | G | 0.03 | 0.029 | 0.020 | 0.61 | 0.20-1.81 | 0.37 |
| rs17519832 | 40281751 | G | 0.01 | 0.011 | 0.005 | 0.44 | 0.05-3.64 | 0.45 |
| rs7979341 | 40281806 | A | 0.33 | 0.315 | 0.410 | 1.55 | 1.12-2.14 | 0.0076 |
| rs112859457 | 40283045 | delA | 0.01 | 0.010 | 0.010 | 0.80 | 0.16-3.96 | 0.78 |
| rs17466080 | 40283045 | T | 0.11 | 0.110 | 0.116 | 1.00 | 0.63-1.59 | 1.00 |
| rs11175852 | 40283206 | A | 0.12 | 0.124 | 0.111 | 0.79 | 0.48-1.29 | 0.35 |
| rs73108368 | 40283782 | T | 0.10 | 0.100 | 0.076 | 0.64 | 0.35-1.17 | 0.15 |
| rs36212067 | 40283797 | G | 0.03 | 0.031 | 0.025 | 0.83 | 0.31-2.26 | 0.72 |
| rs36220738 | 40283853 | T | 0.03 | 0.031 | 0.025 | 0.83 | 0.31-2.26 | 0.72 |
| rs55933352 | 40284262 | dupT | 0.45 | 0.444 | 0.481 | 1.36 | 0.96-1.92 | 0.087 |
| rs147015277 | 40284286 | C | 0.03 | 0.031 | 0.025 | 0.83 | 0.31-2.26 | 0.72 |
| rs34166423 | 40284476 | delTT | 0.25 | 0.254 | 0.200 | 0.80 | 0.55-1.17 | 0.25 |
| rs17519846 | 40284539 | T | 0.39 | 0.396 | 0.310 | 0.73 | 0.52-1.02 | 0.062 |
| rs58793646 | 40284860 | C | 0.01 | 0.012 | 0.005 | 0.42 | 0.05-3.42 | 0.42 |
| rs143262651 | 40285307 | dupTA | 0.01 | 0.012 | 0.020 | 1.47 | 0.46-4.76 | 0.52 |
| rs17466150 | 40285452 | A | 0.14 | 0.142 | 0.110 | 0.74 | 0.46-1.20 | 0.22 |
| rs11175862 | 40285467 | A | 0.25 | 0.254 | 0.200 | 0.80 | 0.55-1.17 | 0.25 |
| rs116911375 | 40285586 | C | 0.01 | 0.011 | 0.010 | 0.95 | 0.20-4.58 | 0.95 |
| rs4310676 | 40286069 | A | 0.33 | 0.315 | 0.410 | 1.55 | 1.12-2.14 | 0.0076 |
| rs7955967 | 40287012 | T | 0.03 | 0.030 | 0.060 | 2.24 | 1.08-4.64 | 0.029 |
| rs11564150 | 40287115 | C | 0.12 | 0.125 | 0.117 | 0.98 | 0.62-1.54 | 0.93 |
| rs4318033 | 40287139 | G | 0.11 | 0.115 | 0.096 | 0.72 | 0.42-1.21 | 0.21 |
| rs4473003 | 40287255 | C | 0.14 | 0.134 | 0.205 | 1.70 | 1.14-2.52 | 0.0090 |
| rs66810434 | 40287551 | dupA | 0.12 | 0.123 | 0.111 | 0.79 | 0.48-1.30 | 0.35 |
| rs11564270 | 40287684 | G | 0.09 | 0.087 | 0.071 | 0.83 | 0.46-1.49 | 0.54 |
| rs11175882 | 40288346 | A | 0.02 | 0.014 | 0.020 | 1.09 | 0.34-3.42 | 0.89 |
| rs17519881 | 40288395 | G | 0.13 | 0.133 | 0.141 | 1.00 | 0.65-1.54 | 1.00 |
| rs17466178 | 40288788 | T | 0.12 | 0.126 | 0.118 | 0.99 | 0.63-1.56 | 0.96 |
| rs78864317 | 40288971 | G | 0.01 | 0.010 | 0.010 | 0.80 | 0.16-3.94 | 0.78 |
| rs138250350 | 40289542 | C | 0.02 | 0.016 | 0.010 | 0.69 | 0.17-2.76 | 0.60 |
| rs74078731 | 40289796 | T | 0.01 | 0.012 | 0.005 | 0.42 | 0.05-3.42 | 0.42 |
| rs111585307 | 40290652 | C | 0.02 | 0.021 | 0.025 | 1.47 | 0.53-4.06 | 0.46 |
| rs17466185 | 40290820 | G | 0.12 | 0.122 | 0.135 | 1.04 | 0.67-1.62 | 0.86 |
| rs11175902 | 40291078 | C | 0.10 | 0.102 | 0.071 | 0.58 | 0.31-1.08 | 0.088 |
| rs7953822 | 40291358 | G | 0.03 | 0.030 | 0.060 | 2.24 | 1.08-4.64 | 0.029 |
| rs76033741 | 40291483 | T | 0.09 | 0.089 | 0.071 | 0.82 | 0.46-1.47 | 0.51 |
| rs76339482 | 40292025 | T | 0.01 | 0.013 | 0.025 | 2.63 | 0.87-7.93 | 0.086 |
| rs56325408 | 40292044 | T | 0.10 | 0.100 | 0.076 | 0.64 | 0.35-1.17 | 0.14 |
| rs7957754 | 40292236 | T | 0.39 | 0.397 | 0.310 | 0.73 | 0.52-1.02 | 0.061 |
| rs60242724 | 40292362 | G | 0.01 | 0.012 | 0.005 | 0.42 | 0.05-3.42 | 0.42 |
| rs199943607 | 40292505 | delTAT | 0.03 | 0.031 | 0.025 | 0.83 | 0.31-2.26 | 0.72 |
| rs7973479 | 40292521 | C | 0.39 | 0.397 | 0.310 | 0.73 | 0.52-1.02 | 0.061 |
| rs73268078 | 40292622 | T | 0.01 | 0.012 | 0.005 | 0.42 | 0.05-3.42 | 0.42 |
| rs11175911 | 40292929 | G | 0.39 | 0.397 | 0.310 | 0.73 | 0.52-1.02 | 0.061 |
| rs12369882 | 40293043 | G | 0.39 | 0.397 | 0.310 | 0.73 | 0.52-1.02 | 0.061 |
| rs149289761 | 40293111 | delA | 0.02 | 0.014 | 0.020 | 1.09 | 0.34-3.42 | 0.89 |
| rs11175913 | 40293233 | T | 0.11 | 0.115 | 0.096 | 0.72 | 0.42-1.21 | 0.21 |
| rs57315777 | 40293294 | T | 0.01 | 0.012 | 0.005 | 0.42 | 0.05-3.42 | 0.42 |
| rs73268087 | 40293480 | T | 0.01 | 0.012 | 0.005 | 0.42 | 0.05-3.42 | 0.42 |
| rs4483664 | 40293852 | A | 0.33 | 0.315 | 0.410 | 1.55 | 1.12-2.14 | 0.0076 |
| rs72446556 | 40294133 | delTATC | 0.48 | 0.480 | 0.500 | 1.17 | 0.78-1.74 | 0.45 |
| rs12820920 | 40294279 | G | 0.39 | 0.397 | 0.310 | 0.73 | 0.52-1.02 | 0.061 |
| rs13377911 | 40294420 | G | 0.01 | 0.012 | 0.020 | 1.47 | 0.46-4.76 | 0.52 |
| rs80025844 | 40294428 | G | 0.01 | 0.012 | 0.020 | 1.47 | 0.46-4.76 | 0.52 |
| rs75802691 | 40294519 | T | 0.02 | 0.014 | 0.020 | 1.09 | 0.34-3.42 | 0.89 |
| rs4768226 | 40294695 | T | 0.03 | 0.031 | 0.025 | 0.83 | 0.31-2.26 | 0.72 |
| rs12314692 | 40295119 | G | 0.11 | 0.115 | 0.096 | 0.72 | 0.42-1.21 | 0.21 |
| rs11175922 | 40295786 | T | 0.39 | 0.397 | 0.315 | 0.74 | 0.53-1.03 | 0.079 |
| rs73102746 | 40296087 | C | 0.12 | 0.121 | 0.118 | 0.95 | 0.60-1.51 | 0.83 |
| rs10784497 | 40296180 | C | 0.11 | 0.115 | 0.096 | 0.72 | 0.42-1.21 | 0.21 |
| rs7957057 | 40296904 | C | 0.32 | 0.313 | 0.399 | 1.51 | 1.09-2.10 | 0.014 |
| rs75468118 | 40296964 | T | 0.01 | 0.012 | 0.020 | 1.47 | 0.46-4.76 | 0.52 |
| rs76607433 | 40297068 | delTT | 0.11 | 0.115 | 0.096 | 0.72 | 0.42-1.21 | 0.22 |
| rs11175926 | 40297120 | A | 0.02 | 0.014 | 0.020 | 1.09 | 0.34-3.42 | 0.89 |
| rs28365224 | 40297142 | A | 0.10 | 0.100 | 0.080 | 0.69 | 0.38-1.24 | 0.22 |
| rs151219680 | 40298711 | A | 0.01 | 0.009 | 0.015 | 1.90 | 0.48-7.52 | 0.36 |
| rs7962011 | 40298741 | T | 0.39 | 0.401 | 0.318 | 0.75 | 0.54-1.04 | 0.085 |
| rs56701518 | 40298921 | G | 0.01 | 0.012 | 0.010 | 0.94 | 0.20-4.51 | 0.94 |
| rs11175934 | 40299999 | G | 0.18 | 0.179 | 0.190 | 1.09 | 0.73-1.61 | 0.68 |
| rs11175935 | 40300004 | T | 0.18 | 0.179 | 0.190 | 1.09 | 0.73-1.61 | 0.68 |
| rs17491061 | 40300216 | C | 0.14 | 0.146 | 0.111 | 0.74 | 0.46-1.20 | 0.23 |
| rs11175941 | 40300597 | A | 0.11 | 0.113 | 0.080 | 0.60 | 0.33-1.07 | 0.081 |
| rs10878340 | 40301293 | A | 0.47 | 0.465 | 0.480 | 1.20 | 0.89-1.63 | 0.24 |
| rs17519950 | 40301386 | T | 0.01 | 0.013 | 0.020 | 1.89 | 0.57-6.20 | 0.30 |
| rs11175943 | 40301974 | C | 0.36 | 0.361 | 0.374 | 1.14 | 0.82-1.57 | 0.44 |
| rs35124455 | 40301989 | G | 0.24 | 0.240 | 0.205 | 0.88 | 0.60-1.29 | 0.52 |
| rs7312696 | 40302049 | C | 0.03 | 0.031 | 0.060 | 2.19 | 1.06-4.50 | 0.034 |
| rs17491082 | 40303070 | C | 0.01 | 0.010 | 0.010 | 0.80 | 0.16-3.94 | 0.78 |
| rs10878341 | 40303675 | T | 0.43 | 0.430 | 0.389 | 0.76 | 0.55-1.06 | 0.11 |
| rs72546323 | 40303859 | A | 0.02 | 0.017 | 0.025 | 1.25 | 0.44-3.54 | 0.68 |
| rs11564269 | 40304620 | T | 0.02 | 0.019 | 0.015 | 0.94 | 0.27-3.30 | 0.92 |
| rs10784499 | 40304688 | T | 0.42 | 0.426 | 0.380 | 0.73 | 0.53-1.02 | 0.067 |
| rs17484286 | 40304884 | G | 0.08 | 0.073 | 0.105 | 1.62 | 0.95-2.77 | 0.079 |
| rs146071397 | 40306304 | delTT | 0.11 | 0.111 | 0.108 | 0.96 | 0.60-1.55 | 0.87 |
| rs10878343 | 40306782 | T | 0.19 | 0.194 | 0.200 | 1.06 | 0.72-1.55 | 0.78 |
| rs146906071 | 40307154 | A | 0.03 | 0.031 | 0.025 | 0.83 | 0.31-2.26 | 0.72 |
| rs11564180 | 40307469 | T | 0.12 | 0.122 | 0.130 | 1.00 | 0.64-1.57 | 0.99 |
| rs11175958 | 40307586 | A | 0.19 | 0.194 | 0.200 | 1.06 | 0.72-1.55 | 0.78 |
| rs201473630 | 40307782 | delT | 0.48 | 0.434 | 0.466 | 1.57 | 1.08-2.28 | 0.019 |
| rs113772607 | 40308029 | T | 0.12 | 0.122 | 0.130 | 1.00 | 0.64-1.57 | 0.99 |
| rs11564149 | 40308362 | T | 0.12 | 0.122 | 0.130 | 1.00 | 0.64-1.57 | 0.99 |
| rs11175963 | 40308969 | T | 0.09 | 0.092 | 0.075 | 0.70 | 0.38-1.27 | 0.24 |
| rs7305344 | 40309044 | A | 0.33 | 0.317 | 0.400 | 1.49 | 1.08-2.06 | 0.016 |
| rs200829235 | 40309245 | delT | 0.01 | 0.016 | 0.005 | 0.34 | 0.04-2.66 | 0.31 |
| rs11175966 | 40309350 | A | 0.19 | 0.194 | 0.200 | 1.05 | 0.72-1.55 | 0.79 |
| rs10784501 | 40310063 | A | 0.45 | 0.440 | 0.470 | 1.43 | 1.04-1.95 | 0.026 |
| rs41286474 | 40310755 | C | 0.12 | 0.121 | 0.119 | 0.93 | 0.59-1.48 | 0.77 |
| rs34073574 | 40311347 | dupT | 0.33 | 0.317 | 0.400 | 1.49 | 1.08-2.06 | 0.016 |
| rs2069228 | 40311356 | T | 0.33 | 0.317 | 0.400 | 1.49 | 1.08-2.06 | 0.016 |
| rs10784503 | 40311898 | C | 0.45 | 0.440 | 0.470 | 1.43 | 1.04-1.95 | 0.026 |
| rs36111759 | 40311965 | delT | 0.46 | 0.453 | 0.465 | 1.40 | 1.02-1.91 | 0.037 |
| rs7136481 | 40312312 | C | 0.01 | 0.011 | 0.015 | 1.07 | 0.29-3.99 | 0.92 |
| rs2896975 | 40312573 | G | 0.33 | 0.317 | 0.400 | 1.49 | 1.08-2.06 | 0.016 |
| rs7302503 | 40312729 | G | 0.33 | 0.317 | 0.400 | 1.49 | 1.08-2.06 | 0.016 |
| rs7137160 | 40312802 | T | 0.45 | 0.440 | 0.470 | 1.43 | 1.04-1.95 | 0.026 |
| rs142435192 | 40312939 | delAGAGATA | 0.12 | 0.121 | 0.119 | 0.93 | 0.59-1.48 | 0.77 |
| rs141885040 | 40313003 | C | 0.01 | 0.011 | 0.015 | 1.16 | 0.31-4.38 | 0.83 |
| rs12427245 | 40313311 | A | 0.03 | 0.031 | 0.025 | 0.83 | 0.31-2.26 | 0.72 |
| rs10467144 | 40313399 | A | 0.45 | 0.440 | 0.470 | 1.43 | 1.04-1.95 | 0.026 |
| rs10784504 | 40313594 | T | 0.45 | 0.440 | 0.470 | 1.43 | 1.04-1.95 | 0.026 |
| rs17443909 | 40313608 | C | 0.01 | 0.016 | 0.005 | 0.34 | 0.04-2.66 | 0.31 |
| rs57421387 | 40313688 | dupT | 0.24 | 0.242 | 0.200 | 0.84 | 0.57-1.25 | 0.39 |
| rs12423419 | 40313699 | G | 0.02 | 0.014 | 0.020 | 1.55 | 0.47-5.11 | 0.48 |
| rs12302298 | 40313846 | G | 0.02 | 0.025 | 0.023 | 0.97 | 0.35-2.66 | 0.95 |
| rs33958906 | 40314059 | T | 0.02 | 0.018 | 0.010 | 0.60 | 0.13-2.73 | 0.51 |
| rs72547977 | 40314520 | A | 0.01 | 0.016 | 0.005 | 0.34 | 0.04-2.66 | 0.31 |
| rs77516464 | 40314612 | A | 0.01 | 0.011 | 0.015 | 1.16 | 0.31-4.38 | 0.83 |
| rs7973058 | 40315043 | T | 0.24 | 0.240 | 0.200 | 0.86 | 0.58-1.26 | 0.44 |
| rs1427267 | 40315090 | T | 0.33 | 0.317 | 0.400 | 1.49 | 1.08-2.06 | 0.016 |
| rs1427266 | 40315120 | C | 0.45 | 0.440 | 0.470 | 1.43 | 1.04-1.95 | 0.026 |
| rs721713 | 40315383 | A | 0.45 | 0.440 | 0.470 | 1.43 | 1.04-1.95 | 0.026 |
| rs721712 | 40315516 | G | 0.45 | 0.440 | 0.470 | 1.43 | 1.04-1.95 | 0.026 |
| rs721711 | 40315526 | G | 0.45 | 0.440 | 0.470 | 1.43 | 1.04-1.95 | 0.026 |
| rs721709 | 40315611 | T | 0.33 | 0.317 | 0.400 | 1.49 | 1.08-2.06 | 0.016 |
| rs5797666 | 40315807 | dupT | 0.33 | 0.317 | 0.400 | 1.49 | 1.08-2.06 | 0.016 |
| rs4767969 | 40315822 | G | 0.45 | 0.440 | 0.470 | 1.43 | 1.04-1.95 | 0.026 |
| rs10878356 | 40316155 | A | 0.19 | 0.192 | 0.195 | 1.03 | 0.70-1.52 | 0.86 |
| rs34567498 | 40317252 | G | 0.02 | 0.014 | 0.020 | 1.55 | 0.47-5.11 | 0.48 |
| rs10784506 | 40317268 | C | 0.46 | 0.449 | 0.465 | 1.41 | 1.03-1.93 | 0.031 |
| rs10878358 | 40317289 | G | 0.46 | 0.449 | 0.465 | 1.41 | 1.03-1.93 | 0.031 |
| rs7132073 | 40317292 | T | 0.12 | 0.130 | 0.123 | 0.99 | 0.63-1.55 | 0.95 |
| rs4768228 | 40317645 | T | 0.03 | 0.031 | 0.025 | 0.83 | 0.31-2.26 | 0.72 |
| rs78774467 | 40317832 | T | 0.02 | 0.024 | 0.025 | 1.19 | 0.44-3.22 | 0.73 |
| rs1427265 | 40318196 | C | 0.45 | 0.440 | 0.470 | 1.43 | 1.04-1.95 | 0.026 |
| rs79847124 | 40318282 | G | 0.02 | 0.014 | 0.020 | 1.55 | 0.47-5.11 | 0.48 |
| rs1427264 | 40318391 | G | 0.45 | 0.440 | 0.470 | 1.43 | 1.04-1.95 | 0.026 |
| rs17466339 | 40318557 | T | 0.17 | 0.177 | 0.147 | 0.78 | 0.51-1.21 | 0.27 |
| rs2114569 | 40318773 | A | 0.45 | 0.440 | 0.470 | 1.43 | 1.04-1.95 | 0.026 |
| rs2114568 | 40318818 | G | 0.45 | 0.440 | 0.470 | 1.43 | 1.04-1.95 | 0.026 |
| rs2162469 | 40319201 | G | 0.45 | 0.440 | 0.470 | 1.43 | 1.04-1.95 | 0.026 |
| rs10784509 | 40319398 | G | 0.45 | 0.440 | 0.470 | 1.43 | 1.04-1.95 | 0.026 |
| rs17466360 | 40319417 | T | 0.03 | 0.030 | 0.025 | 0.75 | 0.28-2.01 | 0.56 |
| rs1896254 | 40319758 | T | 0.45 | 0.440 | 0.470 | 1.43 | 1.04-1.95 | 0.026 |
| rs5797667 | 40319785 | dupA | 0.45 | 0.440 | 0.470 | 1.42 | 1.04-1.94 | 0.026 |
| rs1896253 | 40319793 | T | 0.45 | 0.440 | 0.470 | 1.43 | 1.04-1.95 | 0.026 |
| rs1896252 | 40319957 | T | 0.45 | 0.440 | 0.470 | 1.43 | 1.04-1.95 | 0.026 |
| rs1427263 | 40320032 | C | 0.33 | 0.317 | 0.400 | 1.49 | 1.08-2.06 | 0.016 |
| rs11564205 | 40320207 | G | 0.16 | 0.165 | 0.130 | 0.75 | 0.48-1.17 | 0.20 |
| rs10748030 | 40320386 | G | 0.45 | 0.440 | 0.470 | 1.43 | 1.04-1.95 | 0.026 |
| rs10748031 | 40320464 | T | 0.45 | 0.440 | 0.470 | 1.43 | 1.04-1.95 | 0.026 |
| rs10748032 | 40320465 | G | 0.45 | 0.440 | 0.470 | 1.43 | 1.04-1.95 | 0.026 |
| rs11564177 | 40320530 | C | 0.16 | 0.163 | 0.120 | 0.70 | 0.44-1.10 | 0.12 |
| rs10748033 | 40320593 | C | 0.45 | 0.440 | 0.470 | 1.43 | 1.04-1.95 | 0.026 |
| rs10735931 | 40320699 | T | 0.45 | 0.440 | 0.470 | 1.43 | 1.04-1.95 | 0.026 |
| rs10748034 | 40320816 | C | 0.45 | 0.440 | 0.470 | 1.43 | 1.04-1.95 | 0.026 |
| rs10878368 | 40320983 | A | 0.45 | 0.440 | 0.470 | 1.43 | 1.04-1.95 | 0.026 |
| rs7307276 | 40321211 | T | 0.45 | 0.440 | 0.470 | 1.43 | 1.04-1.95 | 0.026 |
| rs17444028 | 40321281 | A | 0.03 | 0.031 | 0.025 | 0.83 | 0.31-2.26 | 0.72 |
| rs4768229 | 40321446 | T | 0.45 | 0.440 | 0.470 | 1.43 | 1.04-1.95 | 0.026 |
| rs11422410 | 40321509 | delA | 0.45 | 0.442 | 0.470 | 1.42 | 1.04-1.94 | 0.028 |
| rs11564204 | 40321575 | A | 0.16 | 0.163 | 0.120 | 0.70 | 0.44-1.10 | 0.12 |
| rs7137665 | 40322213 | C | 0.33 | 0.317 | 0.400 | 1.49 | 1.08-2.06 | 0.016 |
| rs10878371 | 40322458 | T | 0.45 | 0.440 | 0.470 | 1.43 | 1.04-1.95 | 0.026 |
| rs17444054 | 40322632 | G | 0.03 | 0.031 | 0.025 | 0.83 | 0.31-2.26 | 0.72 |
| rs11176022 | 40322672 | C | 0.09 | 0.092 | 0.075 | 0.70 | 0.38-1.27 | 0.24 |
| rs60750674 | 40322835 | delAT | 0.16 | 0.165 | 0.130 | 0.75 | 0.48-1.17 | 0.20 |
| rs17484342 | 40323341 | A | 0.04 | 0.039 | 0.040 | 1.08 | 0.48-2.43 | 0.86 |
| rs17466430 | 40324198 | A | 0.14 | 0.147 | 0.101 | 0.64 | 0.38-1.06 | 0.081 |
| rs11176030 | 40324651 | T | 0.24 | 0.244 | 0.205 | 0.87 | 0.59-1.28 | 0.48 |
| rs17444068 | 40324973 | C | 0.01 | 0.015 | 0.010 | 0.74 | 0.16-3.37 | 0.70 |
| rs3747891 | 40325081 | C | 0.11 | 0.116 | 0.100 | 0.76 | 0.45-1.26 | 0.28 |
| rs7298930 | 40325698 | A | 0.45 | 0.440 | 0.470 | 1.43 | 1.04-1.95 | 0.026 |
| rs17444089 | 40326004 | C | 0.11 | 0.111 | 0.109 | 0.95 | 0.59-1.52 | 0.83 |
| rs10878377 | 40326202 | T | 0.33 | 0.318 | 0.400 | 1.49 | 1.08-2.06 | 0.015 |
| rs12316801 | 40326497 | C | 0.11 | 0.116 | 0.100 | 0.75 | 0.45-1.26 | 0.28 |
| rs17444096 | 40326499 | C | 0.14 | 0.148 | 0.096 | 0.62 | 0.37-1.04 | 0.073 |
| rs11564175 | 40327240 | A | 0.16 | 0.163 | 0.120 | 0.70 | 0.44-1.10 | 0.12 |
| rs17444103 | 40327357 | C | 0.01 | 0.013 | 0.005 | 0.40 | 0.05-3.23 | 0.39 |
| rs2404832 | 40328650 | G | 0.43 | 0.440 | 0.395 | 0.75 | 0.54-1.04 | 0.084 |
| rs2404833 | 40328655 | G | 0.03 | 0.031 | 0.060 | 2.21 | 1.07-4.56 | 0.032 |
| rs11564267 | 40328922 | T | 0.16 | 0.165 | 0.125 | 0.71 | 0.45-1.13 | 0.15 |
| rs17520244 | 40329068 | delG | 0.16 | 0.165 | 0.125 | 0.71 | 0.45-1.13 | 0.15 |
| rs12370996 | 40329369 | T | 0.02 | 0.024 | 0.005 | 0.22 | 0.03-1.69 | 0.15 |
| rs10506152 | 40329673 | A | 0.12 | 0.122 | 0.114 | 0.96 | 0.60-1.53 | 0.86 |
| rs12322674 | 40329743 | G | 0.01 | 0.011 | 0.015 | 1.07 | 0.29-3.99 | 0.92 |
| rs11176053 | 40330228 | T | 0.31 | 0.321 | 0.265 | 0.71 | 0.49-1.02 | 0.064 |
| rs17444124 | 40330692 | C | 0.40 | 0.405 | 0.330 | 0.76 | 0.55-1.05 | 0.096 |
| rs140812745 | 40331456 | C | 0.01 | 0.013 | 0.005 | 0.40 | 0.05-3.23 | 0.39 |
| rs116447010 | 40331479 | G | 0.01 | 0.010 | 0.010 | 1.41 | 0.29-6.71 | 0.67 |
| rs59493060 | 40331517 | T | 0.01 | 0.013 | 0.010 | 0.85 | 0.18-4.00 | 0.84 |
| rs11461395 | 40331598 | delA | 0.35 | 0.343 | 0.433 | 1.46 | 1.06-2.01 | 0.021 |
| rs73275761 | 40331791 | G | 0.16 | 0.164 | 0.121 | 0.70 | 0.44-1.11 | 0.13 |
| rs17444145 | 40332078 | T | 0.12 | 0.117 | 0.111 | 0.99 | 0.61-1.61 | 0.97 |
| rs10878386 | 40332126 | G | 0.09 | 0.097 | 0.075 | 0.68 | 0.37-1.23 | 0.20 |
| rs17466521 | 40332765 | G | 0.28 | 0.285 | 0.260 | 0.83 | 0.59-1.17 | 0.29 |
| rs12367542 | 40332845 | T | 0.24 | 0.239 | 0.200 | 0.86 | 0.58-1.26 | 0.44 |
| rs17520278 | 40332946 | G | 0.16 | 0.167 | 0.131 | 0.74 | 0.47-1.16 | 0.19 |
| rs35031086 | 40332961 | delA | 0.20 | 0.194 | 0.267 | 1.45 | 0.98-2.14 | 0.060 |
| rs28365229 | 40333343 | T | 0.03 | 0.028 | 0.025 | 0.91 | 0.33-2.53 | 0.86 |
| rs17491417 | 40333667 | T | 0.01 | 0.013 | 0.010 | 0.85 | 0.18-3.99 | 0.84 |
| rs2896976 | 40334122 | A | 0.03 | 0.030 | 0.060 | 2.24 | 1.08-4.64 | 0.029 |
| rs4465428 | 40334389 | C | 0.13 | 0.121 | 0.195 | 1.82 | 1.22-2.72 | 0.0036 |
| rs61007767 | 40334496 | G | 0.17 | 0.169 | 0.136 | 0.77 | 0.49-1.20 | 0.24 |
| rs60776971 | 40334626 | C | 0.16 | 0.165 | 0.130 | 0.74 | 0.47-1.16 | 0.19 |
| rs149596967 | 40334824 | T | 0.01 | 0.013 | 0.005 | 0.40 | 0.05-3.23 | 0.39 |
| rs7302841 | 40335853 | A | 0.28 | 0.291 | 0.235 | 0.78 | 0.55-1.12 | 0.18 |
| rs11356084 | 40337134 | delA | 0.49 | 0.409 | 0.481 | 1.55 | 1.13-2.12 | 0.0065 |
| rs10506154 | 40337211 | G | 0.03 | 0.028 | 0.025 | 0.91 | 0.33-2.53 | 0.86 |
| rs715403 | 40337381 | C | 0.31 | 0.308 | 0.318 | 1.03 | 0.74-1.43 | 0.87 |
| rs715402 | 40337412 | G | 0.13 | 0.122 | 0.205 | 1.91 | 1.28-2.84 | 0.0015 |
| rs6581667 | 40337704 | C | 0.50 | 0.400 | 0.482 | 1.61 | 1.17-2.20 | 0.0032 |
| rs6581668 | 40337804 | A | 0.49 | 0.409 | 0.481 | 1.55 | 1.13-2.12 | 0.0065 |
| rs17491466 | 40338051 | T | 0.02 | 0.024 | 0.025 | 1.19 | 0.44-3.22 | 0.73 |
| rs17491473 | 40338065 | C | 0.01 | 0.013 | 0.015 | 1.04 | 0.28-3.86 | 0.96 |
| rs200521371 | 40338158 | delCTC | 0.10 | 0.098 | 0.075 | 0.66 | 0.36-1.20 | 0.17 |
| rs4767970 | 40338159 | C | 0.20 | 0.184 | 0.295 | 1.78 | 1.28-2.49 | 0.00066 |
| rs4767972 | 40338321 | C | 0.18 | 0.173 | 0.270 | 1.82 | 1.26-2.62 | 0.0013 |
| rs1427271 | 40338592 | T | 0.18 | 0.171 | 0.265 | 1.78 | 1.24-2.57 | 0.0019 |
| rs1427272 | 40338678 | G | 0.18 | 0.173 | 0.270 | 1.82 | 1.26-2.62 | 0.0013 |
| rs1427273 | 40338928 | C | 0.18 | 0.173 | 0.270 | 1.82 | 1.26-2.62 | 0.0013 |
| rs10732751 | 40339280 | A | 0.18 | 0.173 | 0.270 | 1.82 | 1.26-2.62 | 0.0013 |
| rs12306060 | 40339762 | T | 0.18 | 0.173 | 0.270 | 1.82 | 1.26-2.62 | 0.0013 |
| rs1365763 | 40340781 | T | 0.18 | 0.172 | 0.270 | 1.84 | 1.28-2.65 | 0.0011 |
| rs79106392 | 40340999 | G | 0.01 | 0.011 | 0.015 | 1.07 | 0.29-3.99 | 0.92 |
| rs7963697 | 40341114 | C | 0.18 | 0.173 | 0.270 | 1.82 | 1.26-2.62 | 0.0013 |
| rs11316380 | 40341175 | delG | 0.28 | 0.288 | 0.235 | 0.78 | 0.55-1.11 | 0.16 |
| rs7963086 | 40341367 | G | 0.13 | 0.123 | 0.205 | 1.88 | 1.26-2.81 | 0.0019 |
| rs7956787 | 40341962 | T | 0.18 | 0.173 | 0.270 | 1.82 | 1.26-2.62 | 0.0013 |
| rs7956898 | 40342002 | T | 0.18 | 0.173 | 0.270 | 1.82 | 1.26-2.62 | 0.0013 |
| rs7954061 | 40342208 | A | 0.18 | 0.173 | 0.270 | 1.82 | 1.26-2.62 | 0.0013 |
| rs7957151 | 40342241 | T | 0.18 | 0.173 | 0.270 | 1.81 | 1.26-2.61 | 0.0014 |
| rs369369244 | 40342435 | T | 0.03 | 0.032 | 0.015 | 0.47 | 0.14-1.54 | 0.21 |
| rs919175 | 40342603 | C | 0.18 | 0.173 | 0.270 | 1.81 | 1.26-2.61 | 0.0014 |
| rs1035812 | 40342980 | C | 0.18 | 0.173 | 0.270 | 1.81 | 1.26-2.61 | 0.0014 |
| rs1365764 | 40343200 | G | 0.18 | 0.173 | 0.270 | 1.81 | 1.26-2.61 | 0.0014 |
| rs58392855 | 40343258 | - | 0.18 | 0.174 | 0.270 | 1.80 | 1.25-2.59 | 0.0016 |
| rs17461964 | 40343273 | C | 0.18 | 0.174 | 0.270 | 1.80 | 1.25-2.59 | 0.0016 |
| rs10784518 | 40343313 | G | 0.18 | 0.174 | 0.270 | 1.80 | 1.25-2.59 | 0.0016 |
| rs4768232 | 40343438 | C | 0.18 | 0.174 | 0.270 | 1.80 | 1.25-2.59 | 0.0016 |
| rs11176118 | 40343669 | A | 0.18 | 0.178 | 0.182 | 1.05 | 0.70-1.57 | 0.83 |
| rs3943893 | 40344001 | A | 0.18 | 0.174 | 0.270 | 1.80 | 1.25-2.59 | 0.0016 |
| rs2162471 | 40344030 | C | 0.18 | 0.178 | 0.182 | 1.05 | 0.70-1.57 | 0.83 |
| rs1035813 | 40344106 | C | 0.18 | 0.174 | 0.270 | 1.80 | 1.25-2.59 | 0.0016 |
| rs75846146 | 40344488 | A | 0.01 | 0.010 | 0.010 | 1.41 | 0.29-6.71 | 0.67 |
| rs10459265 | 40344501 | A | 0.50 | 0.400 | 0.486 | 1.59 | 1.16-2.18 | 0.0037 |
| rs11832935 | 40345619 | A | 0.50 | 0.400 | 0.486 | 1.59 | 1.16-2.18 | 0.0037 |
| rs17520445 | 40345687 | T | 0.01 | 0.013 | 0.015 | 1.04 | 0.28-3.86 | 0.96 |
| rs11564147 | 40345691 | A | 0.10 | 0.107 | 0.060 | 0.58 | 0.31-1.07 | 0.083 |
| rs10715759 | 40345827 | delA | 0.28 | 0.288 | 0.235 | 0.78 | 0.55-1.11 | 0.16 |
| rs7296657 | 40345925 | C | 0.18 | 0.174 | 0.270 | 1.80 | 1.25-2.59 | 0.0016 |
| rs7312497 | 40346050 | A | 0.18 | 0.174 | 0.270 | 1.80 | 1.25-2.59 | 0.0016 |
| rs10715758 | 40346188 | dupT | 0.18 | 0.173 | 0.273 | 1.83 | 1.27-2.64 | 0.0012 |
| rs148319899 | 40346421 | C | 0.03 | 0.028 | 0.025 | 0.91 | 0.33-2.53 | 0.86 |
| rs10784522 | 40346563 | T | 0.28 | 0.288 | 0.235 | 0.78 | 0.55-1.11 | 0.16 |
| rs33995883 | 40346884 | G | 0.01 | 0.013 | 0.005 | 0.40 | 0.05-3.23 | 0.39 |
| rs11289057 | 40347027 | dupT | 0.18 | 0.174 | 0.270 | 1.80 | 1.25-2.59 | 0.0016 |
| rs74467833 | 40347033 | G | 0.31 | 0.307 | 0.318 | 1.04 | 0.74-1.45 | 0.82 |
| rs17484493 | 40347071 | C | 0.03 | 0.028 | 0.025 | 0.91 | 0.33-2.53 | 0.86 |
| rs7131694 | 40347145 | A | 0.13 | 0.120 | 0.195 | 1.83 | 1.22-2.74 | 0.0033 |
| rs17520459 | 40347509 | C | 0.01 | 0.010 | 0.010 | 0.80 | 0.16-3.94 | 0.78 |
| rs12311273 | 40347815 | C | 0.28 | 0.288 | 0.235 | 0.78 | 0.55-1.11 | 0.16 |
| rs58911468 | 40347876 | C | 0.12 | 0.128 | 0.090 | 0.71 | 0.42-1.19 | 0.19 |
| rs35847030 | 40347957 | dupA | 0.31 | 0.307 | 0.307 | 0.98 | 0.70-1.38 | 0.93 |
| rs1365765 | 40348019 | A | 0.18 | 0.174 | 0.270 | 1.80 | 1.25-2.59 | 0.0016 |
| rs66513412 | 40348203 | delT | 0.47 | 0.478 | 0.383 | 0.71 | 0.52-0.96 | 0.028 |
| rs2896977 | 40348203 | C | 0.34 | 0.337 | 0.332 | 0.97 | 0.70-1.34 | 0.84 |
| rs199606989 | 40348204 | C | 0.47 | 0.478 | 0.383 | 0.71 | 0.52-0.96 | 0.028 |
| rs1365766 | 40348271 | C | 0.18 | 0.174 | 0.270 | 1.80 | 1.25-2.59 | 0.0016 |
| rs187998728 | 40348859 | T | 0.02 | 0.020 | 0.017 | 0.77 | 0.22-2.76 | 0.69 |
| rs7294958 | 40348887 | T | 0.19 | 0.174 | 0.273 | 1.82 | 1.26-2.62 | 0.0013 |
| rs61915610 | 40348899 | T | 0.31 | 0.307 | 0.320 | 1.05 | 0.75-1.46 | 0.77 |
| rs7294952 | 40349074 | A | 0.19 | 0.174 | 0.273 | 1.82 | 1.26-2.62 | 0.0013 |
| rs78560388 | 40349106 | T | 0.01 | 0.013 | 0.015 | 1.04 | 0.28-3.86 | 0.96 |
| rs61579260 | 40349510 | dupCTT | 0.19 | 0.175 | 0.273 | 1.82 | 1.26-2.62 | 0.0013 |
| rs59096461 | 40349559 | dupTGGAGTGC | 0.19 | 0.174 | 0.273 | 1.82 | 1.26-2.62 | 0.0013 |
| rs59980086 | 40349691 | dupT | 0.31 | 0.307 | 0.320 | 1.05 | 0.75-1.46 | 0.77 |
| rs4768233 | 40349986 | C | 0.19 | 0.174 | 0.273 | 1.82 | 1.26-2.62 | 0.0013 |
| rs4768234 | 40350028 | G | 0.19 | 0.174 | 0.273 | 1.82 | 1.26-2.62 | 0.0013 |
| rs4768235 | 40350284 | A | 0.19 | 0.174 | 0.273 | 1.82 | 1.26-2.62 | 0.0013 |
| rs7314455 | 40350343 | C | 0.19 | 0.174 | 0.273 | 1.82 | 1.26-2.62 | 0.0013 |
| rs7313525 | 40350381 | G | 0.19 | 0.174 | 0.273 | 1.82 | 1.26-2.62 | 0.0013 |
| rs7313895 | 40350592 | G | 0.19 | 0.174 | 0.273 | 1.82 | 1.26-2.62 | 0.0013 |
| rs7314863 | 40350689 | C | 0.19 | 0.174 | 0.273 | 1.82 | 1.26-2.62 | 0.0013 |
| rs12318400 | 40350987 | C | 0.01 | 0.011 | 0.015 | 1.07 | 0.29-3.99 | 0.92 |
| rs17491652 | 40351067 | T | 0.01 | 0.013 | 0.010 | 0.85 | 0.18-4.00 | 0.84 |
| rs12426639 | 40351975 | T | 0.03 | 0.028 | 0.025 | 0.92 | 0.33-2.52 | 0.87 |
| rs11176154 | 40352100 | C | 0.31 | 0.307 | 0.320 | 1.05 | 0.75-1.46 | 0.77 |
| rs7311247 | 40352112 | T | 0.18 | 0.173 | 0.268 | 1.80 | 1.25-2.60 | 0.0017 |
| rs7137437 | 40352153 | C | 0.19 | 0.174 | 0.273 | 1.82 | 1.26-2.62 | 0.0013 |
| rs75439407 | 40352236 | delG | 0.31 | 0.307 | 0.320 | 1.05 | 0.75-1.46 | 0.77 |
| rs73107108 | 40352271 | C | 0.13 | 0.129 | 0.135 | 1.00 | 0.65-1.55 | 1.00 |
| rs76555745 | 40352470 | - | 0.27 | 0.277 | 0.250 | 0.80 | 0.53-1.22 | 0.30 |
| rs11176155 | 40352653 | A | 0.19 | 0.174 | 0.273 | 1.82 | 1.26-2.62 | 0.0013 |
| rs10878410 | 40352874 | C | 0.19 | 0.174 | 0.273 | 1.82 | 1.26-2.62 | 0.0013 |
| rs138382676 | 40352927 | C | 0.13 | 0.130 | 0.128 | 0.97 | 0.62-1.52 | 0.90 |
| rs10878411 | 40353047 | C | 0.19 | 0.180 | 0.280 | 1.84 | 1.28-2.64 | 0.00089 |
| rs11176160 | 40353065 | C | 0.19 | 0.176 | 0.273 | 1.81 | 1.26-2.60 | 0.0014 |
| rs11176161 | 40353080 | G | 0.19 | 0.174 | 0.273 | 1.82 | 1.26-2.62 | 0.0013 |
| rs10878412 | 40353111 | T | 0.19 | 0.174 | 0.273 | 1.82 | 1.26-2.62 | 0.0013 |
| rs10878413 | 40353158 | T | 0.19 | 0.174 | 0.273 | 1.82 | 1.26-2.62 | 0.0013 |
| rs142726158 | 40353226 | C | 0.19 | 0.179 | 0.271 | 1.76 | 1.22-2.55 | 0.0027 |
| rs185723137 | 40353244 | A | 0.19 | 0.179 | 0.271 | 1.76 | 1.22-2.55 | 0.0027 |
| rs61915611 | 40353279 | G | 0.19 | 0.179 | 0.278 | 1.76 | 1.23-2.52 | 0.0022 |
| rs61915612 | 40353283 | A | 0.19 | 0.179 | 0.278 | 1.76 | 1.23-2.52 | 0.0022 |
| rs140722234 | 40353344 | A | 0.18 | 0.173 | 0.265 | 1.79 | 1.23-2.59 | 0.0022 |
| rs191099092 | 40353383 | T | 0.10 | 0.107 | 0.060 | 0.58 | 0.31-1.07 | 0.083 |
| rs113693842 | 40353440 | C | 0.19 | 0.174 | 0.273 | 1.82 | 1.26-2.62 | 0.0013 |
| rs112873815 | 40353442 | A | 0.01 | 0.013 | 0.015 | 1.04 | 0.28-3.86 | 0.96 |
| rs112767992 | 40353467 | T | 0.26 | 0.265 | 0.210 | 0.77 | 0.53-1.11 | 0.16 |
| rs12368227 | 40353632 | T | 0.28 | 0.288 | 0.235 | 0.78 | 0.55-1.11 | 0.16 |
| rs12426326 | 40353633 | A | 0.03 | 0.028 | 0.025 | 0.92 | 0.33-2.52 | 0.87 |
| rs12368229 | 40353660 | A | 0.18 | 0.178 | 0.185 | 1.07 | 0.72-1.60 | 0.73 |
| rs17491673 | 40353731 | T | 0.01 | 0.013 | 0.015 | 1.04 | 0.28-3.86 | 0.96 |
| rs3930031 | 40353732 | G | 0.19 | 0.174 | 0.273 | 1.82 | 1.26-2.62 | 0.0013 |
| rs17484541 | 40353949 | A | 0.03 | 0.028 | 0.025 | 0.92 | 0.33-2.52 | 0.87 |
| rs5017705 | 40353970 | G | 0.19 | 0.174 | 0.273 | 1.82 | 1.26-2.62 | 0.0013 |
| rs7308626 | 40354127 | C | 0.19 | 0.174 | 0.273 | 1.82 | 1.26-2.62 | 0.0013 |
| rs890575 | 40354593 | G | 0.19 | 0.174 | 0.273 | 1.82 | 1.26-2.62 | 0.0013 |
| rs10748040 | 40354745 | A | 0.19 | 0.174 | 0.273 | 1.82 | 1.26-2.62 | 0.0013 |
| rs11835417 | 40354845 | A | 0.02 | 0.018 | 0.047 | 3.97 | 1.66-9.49 | 0.0019 |
| rs139470314 | 40355192 | delG | 0.01 | 0.010 | 0.010 | 0.80 | 0.16-3.94 | 0.78 |
| rs7976715 | 40355348 | A | 0.50 | 0.389 | 0.486 | 1.68 | 1.22-2.31 | 0.0015 |
| rs7976724 | 40355370 | G | 0.19 | 0.174 | 0.273 | 1.82 | 1.26-2.62 | 0.0013 |
| rs7963987 | 40355395 | A | 0.19 | 0.174 | 0.273 | 1.82 | 1.26-2.62 | 0.0013 |
| rs2162472 | 40355416 | G | 0.19 | 0.174 | 0.273 | 1.82 | 1.26-2.62 | 0.0013 |
| rs112905910 | 40355548 | dupTT | 0.19 | 0.175 | 0.278 | 1.85 | 1.28-2.67 | 0.00096 |
| rs182035168 | 40355988 | G | 0.02 | 0.014 | 0.020 | 1.74 | 0.55-5.50 | 0.34 |
| rs11564266 | 40356393 | C | 0.03 | 0.028 | 0.025 | 0.92 | 0.33-2.52 | 0.87 |
| rs34073451 | 40356494 | - | 0.19 | 0.174 | 0.273 | 1.82 | 1.26-2.62 | 0.0013 |
| rs11564173 | 40356669 | A | 0.12 | 0.128 | 0.090 | 0.71 | 0.42-1.19 | 0.19 |
| rs7306545 | 40356747 | T | 0.19 | 0.174 | 0.273 | 1.82 | 1.26-2.62 | 0.0013 |
| rs114705842 | 40357016 | G | 0.01 | 0.010 | 0.010 | 0.80 | 0.16-3.94 | 0.78 |
| rs7306684 | 40357018 | A | 0.19 | 0.175 | 0.270 | 1.82 | 1.27-2.63 | 0.0013 |
| rs112259589 | 40357164 | T | 0.10 | 0.098 | 0.075 | 0.66 | 0.36-1.20 | 0.17 |
| rs73277531 | 40357182 | G | 0.12 | 0.128 | 0.090 | 0.71 | 0.42-1.19 | 0.19 |
| rs77689380 | 40357364 | dupTT | 0.19 | 0.174 | 0.273 | 1.82 | 1.26-2.62 | 0.0013 |
| rs80172174 | 40357572 | T | 0.03 | 0.028 | 0.025 | 0.92 | 0.33-2.52 | 0.87 |
| rs73277533 | 40357725 | A | 0.12 | 0.128 | 0.090 | 0.71 | 0.42-1.19 | 0.19 |
| rs112677925 | 40357832 | A | 0.03 | 0.027 | 0.020 | 0.69 | 0.23-2.07 | 0.50 |
| rs147851429 | 40357909 | delCTACA | 0.01 | 0.010 | 0.010 | 0.80 | 0.16-3.94 | 0.78 |
| rs17462055 | 40357968 | G | 0.18 | 0.178 | 0.185 | 1.07 | 0.72-1.60 | 0.73 |
| rs77668961 | 40358013 | T | 0.05 | 0.052 | 0.035 | 0.61 | 0.26-1.40 | 0.24 |
| rs10784528 | 40358177 | T | 0.13 | 0.123 | 0.205 | 1.85 | 1.24-2.76 | 0.0024 |
| rs35500726 | 40358736 | dupT | 0.13 | 0.123 | 0.205 | 1.85 | 1.24-2.76 | 0.0024 |
| rs10784532 | 40358801 | T | 0.30 | 0.303 | 0.318 | 1.07 | 0.77-1.50 | 0.68 |
| rs79836766 | 40359187 | A | 0.01 | 0.013 | 0.015 | 1.04 | 0.28-3.86 | 0.96 |
| rs79074615 | 40359211 | T | 0.02 | 0.024 | 0.025 | 1.19 | 0.44-3.22 | 0.73 |
| rs3789330 | 40359501 | A | 0.19 | 0.174 | 0.273 | 1.82 | 1.26-2.62 | 0.0013 |
| rs11836288 | 40359972 | T | 0.10 | 0.098 | 0.075 | 0.66 | 0.36-1.20 | 0.17 |
| rs117891010 | 40359994 | C | 0.01 | 0.013 | 0.005 | 0.40 | 0.05-3.23 | 0.39 |
| rs10784536 | 40360610 | A | 0.19 | 0.174 | 0.273 | 1.82 | 1.26-2.62 | 0.0013 |
| rs7971919 | 40360895 | G | 0.19 | 0.174 | 0.273 | 1.82 | 1.26-2.62 | 0.0013 |
| rs7962116 | 40360980 | T | 0.19 | 0.174 | 0.273 | 1.82 | 1.26-2.62 | 0.0013 |
| rs10548450 | 40361243 | dupCATA | 0.19 | 0.174 | 0.273 | 1.82 | 1.26-2.62 | 0.0013 |
| rs17520606 | 40361350 | G | 0.01 | 0.011 | 0.015 | 1.09 | 0.28-4.23 | 0.90 |
| rs1365768 | 40361413 | A | 0.03 | 0.028 | 0.025 | 0.92 | 0.33-2.52 | 0.86 |
| rs1365769 | 40361487 | G | 0.03 | 0.028 | 0.025 | 0.92 | 0.33-2.52 | 0.87 |
| rs2896978 | 40361614 | G | 0.19 | 0.174 | 0.273 | 1.82 | 1.26-2.62 | 0.0013 |
| rs2404836 | 40361674 | A | 0.19 | 0.174 | 0.273 | 1.82 | 1.26-2.62 | 0.0013 |
| rs4767973 | 40362446 | A | 0.36 | 0.345 | 0.450 | 1.57 | 1.15-2.16 | 0.0051 |
| rs4768236 | 40362670 | C | 0.36 | 0.347 | 0.455 | 1.58 | 1.15-2.15 | 0.0043 |
| rs4768237 | 40362885 | T | 0.37 | 0.360 | 0.455 | 1.48 | 1.09-2.01 | 0.013 |
| rs10626458 | 40363313 | - | 0.13 | 0.120 | 0.203 | 1.91 | 1.27-2.88 | 0.0018 |
| rs11317573 | 40363385 | dupT | 0.37 | 0.360 | 0.455 | 1.48 | 1.09-2.01 | 0.013 |
| rs137858615 | 40364290 | A | 0.11 | 0.119 | 0.080 | 0.67 | 0.39-1.15 | 0.15 |
| rs35403247 | 40364517 | dupT | 0.27 | 0.277 | 0.230 | 0.80 | 0.56-1.16 | 0.24 |
| rs11374074 | 40365546 | dupT | 0.50 | 0.419 | 0.485 | 1.45 | 1.06-1.98 | 0.019 |
| rs17444223 | 40365793 | A | 0.19 | 0.188 | 0.200 | 1.09 | 0.74-1.61 | 0.67 |
| rs17491786 | 40365902 | G | 0.01 | 0.010 | 0.010 | 1.41 | 0.29-6.71 | 0.67 |
| rs12426362 | 40366046 | T | 0.37 | 0.360 | 0.455 | 1.48 | 1.09-2.01 | 0.013 |
| rs17466605 | 40366178 | A | 0.37 | 0.360 | 0.455 | 1.48 | 1.09-2.01 | 0.013 |
| rs17491807 | 40366360 | A | 0.01 | 0.012 | 0.010 | 0.80 | 0.17-3.82 | 0.78 |
| rs17466626 | 40366829 | G | 0.02 | 0.024 | 0.005 | 0.19 | 0.02-1.41 | 0.10 |
| rs3789329 | 40366962 | C | 0.04 | 0.039 | 0.025 | 0.59 | 0.22-1.59 | 0.29 |
| rs3789328 | 40367205 | A | 0.37 | 0.363 | 0.460 | 1.49 | 1.09-2.03 | 0.012 |
| rs12426498 | 40367513 | T | 0.09 | 0.098 | 0.070 | 0.60 | 0.32-1.12 | 0.11 |
| rs4768238 | 40367556 | A | 0.14 | 0.132 | 0.220 | 1.90 | 1.29-2.79 | 0.0012 |
| rs66737902 | 40367861 | C | 0.11 | 0.119 | 0.080 | 0.67 | 0.39-1.15 | 0.15 |
| rs1365770 | 40368501 | G | 0.37 | 0.360 | 0.455 | 1.48 | 1.09-2.01 | 0.013 |
| rs367772598 | 40368691 | delT | 0.17 | 0.167 | 0.179 | 1.05 | 0.70-1.58 | 0.81 |
| rs12422278 | 40369211 | A | 0.04 | 0.039 | 0.025 | 0.59 | 0.22-1.59 | 0.29 |
| rs1465528 | 40369814 | C | 0.37 | 0.361 | 0.455 | 1.47 | 1.08-2.00 | 0.014 |
| rs17444285 | 40369850 | T | 0.09 | 0.095 | 0.060 | 0.70 | 0.38-1.29 | 0.25 |
| rs7968048 | 40370171 | C | 0.37 | 0.361 | 0.455 | 1.47 | 1.08-2.00 | 0.014 |
| rs11609433 | 40370967 | G | 0.31 | 0.313 | 0.273 | 0.87 | 0.62-1.22 | 0.43 |
| rs4374003 | 40371801 | C | 0.50 | 0.493 | 0.432 | 0.76 | 0.52-1.09 | 0.14 |
| rs7962370 | 40372127 | A | 0.13 | 0.120 | 0.195 | 1.81 | 1.21-2.70 | 0.0040 |
| rs11176261 | 40372450 | C | 0.09 | 0.097 | 0.070 | 0.61 | 0.33-1.13 | 0.12 |
| rs7137173 | 40373020 | A | 0.19 | 0.186 | 0.192 | 1.05 | 0.70-1.55 | 0.83 |
| rs17444299 | 40373229 | A | 0.02 | 0.019 | 0.025 | 1.44 | 0.51-4.08 | 0.49 |
| rs17444306 | 40373310 | T | 0.12 | 0.122 | 0.120 | 0.95 | 0.59-1.52 | 0.83 |
| rs10467147 | 40373560 | A | 0.29 | 0.296 | 0.263 | 0.88 | 0.62-1.24 | 0.45 |
| rs34852608 | 40373587 | delCTGA | 0.01 | 0.013 | 0.005 | 0.49 | 0.06-3.96 | 0.50 |
| rs11176274 | 40373887 | T | 0.31 | 0.312 | 0.268 | 0.85 | 0.61-1.19 | 0.34 |
| rs4113953 | 40374188 | G | 0.37 | 0.365 | 0.454 | 1.43 | 1.05-1.96 | 0.024 |
| rs5010412 | 40374190 | T | 0.37 | 0.364 | 0.454 | 1.44 | 1.05-1.96 | 0.023 |
| rs4113954 | 40374195 | A | 0.08 | 0.082 | 0.055 | 0.72 | 0.38-1.37 | 0.32 |
| A1: reference allele; MAF: minor allele frequency; Fcontrol: low/moderate risk of ethanol dependence; Fcase: high risk of ethanol dependence; OR: odds ratio; 95% CI: 95% confidence interval; P: p-value (additive model); del: deletion; dup: duplication; ins: insertion | | | | | | | | |
| Multivariate logistic regression - covariates: sex, age and principal component 1 (PC1) | | | | | | | | |
| ^a^Human genome assembly: GRCh38 | | | | | | | | |

| **Table S3. Association of *LRRK2* variants with alcohol dependence in individuals from the US cohort with diferent degrees of European ancestry** | | | | | | | | | | | | | | | | | | |
| --- | --- | --- | --- | --- | --- | --- | --- | --- | --- | --- | --- | --- | --- | --- | --- | --- | --- | --- |
|  |  |  |  | **EUR_AIM_ > Median (n = 658)** | | | | |  | **EUR_AIM_ < Median (n = 658)** | | | | |  | **Meta-analysis** | | |
| **SNP** | **Coordinate^a^** | **A1** |  | **MAF** | **SE** | **OR** | **95% CI** | **P** |  | **MAF** | **SE** | **OR** | **95% CI** | **P** |  | **N** | **OR** | **P_meta_** |
| rs4767971 | chr12:40338230 | C |  | 0.13 | 0.11 | 1.10 | 0.98-1.40 | 0.11 |  | 0.20 | 0.08 | 1.37 | 1.07-1.59 | **0.007** |  | 2 | 1.24 | **0.045** |
| rs4768231 | chr12:40343381 | G |  | 0.16 | 0.11 | 1.12 | 0.98-1.52 | 0.09 |  | 0.31 | 0.08 | 1.43 | 1.10-1.62 | **0.009** |  | 2 | 1.28 | **0.042** |
| rs7307310 | chr12:40351379 | T |  | 0.13 | 0.16 | 0.96 | 0.62-1.32 | 0.45 |  | 0.16 | 0.12 | 1.24 | 0.85-1.54 | 0.27 |  | 2 | 1.11 | 0.20 |
| Only the genotypes of rs4767971, rs4768231 and rs7307310 were available for the United States (NIH) cohort | | | | | | | | | | | | | | | | | | |
| A1: reference allele; SE: SE of odds ratio (OR); 95% CI: 95% confidence interval; P: p-value (additive model); N: number of valid studies; P: p value for random-effects meta-analysis | | | | | | | | | | | | | | | | | | |
| Multivariate logistic regression - covariates: sex and age | | | | | | | | | | | | | | | | | | |
| ^a^Human genome assembly: GRCh38 | | | | | | | | | | | | | | | | | | |
| EUR_AIM_: European ancestry informative marker (AIM) score (median = 0,506) | | | | | | | | | | | | | | | | | | |

| **Table S4. Random-effects meta-analysis of the *LRRK2* variants associated with alcohol dependence in samples from Brazil and United States** | | | | | | | | | | | | | | |
| --- | --- | --- | --- | --- | --- | --- | --- | --- | --- | --- | --- | --- | --- | --- |
|  |  |  | **Bambuí (Brazil) discovery sample** | |  | **Pelotas (Brazil) replication sample** | |  | **United States (NIH) replication sample** | |  | **Meta-analysis** | | |
| **SNP** | **Coordinate^a^** | **A1** | **SE** | **OR** |  | **SE** | **OR** |  | **SE** | **OR** |  | **N** | **OR** | **P** |
| rs4767971 | chr12:40338230 | C | 0.195 | 2.00 |  | 0.156 | 1.36 |  | 0.107 | 1.25 |  | 3 | 1.44 | **4.4x10^-3^** |
| rs4768231 | chr12:40343381 | G | 0.196 | 2.03 |  | 0.138 | 1.33 |  | 0.093 | 1.23 |  | 3 | 1.42 | **6.2x10^-3^** |
| rs7307310 | chr12:40351379 | T | 0.195 | 1.94 |  | 0.157 | 1.44 |  | 0.112 | 1.10 |  | 3 | 1.41 | 0.032 |
| Only the genotypes of rs4767971, rs4768231 and rs7307310 were available for the three cohorts | | | | | | | | | | | | | | |
| A1: reference allele; SE: SE of odds ratio (OR); N: number of valid studies; P: p value for random-effects meta-analysis | | | | | | | | | | | | | | |
| Siginifcance level assumed for this analysis, P < 0.017 | | | | | | | | | | | | | | |
| ^a^Human genome assembly: GRCh38 | | | | | | | | | | | | | | |
